# Impact of photobleaching on quantitative, spatio-temporal, super-resolution imaging of mitochondria in live *C. elegans* larvae

**DOI:** 10.1038/s44303-024-00043-1

**Published:** 2024-11-06

**Authors:** Segos Ioannis, Van Eeckhoven Jens, Greig Alan, Redd Michael, Thrasivoulou Christopher, Conradt Barbara

**Affiliations:** Research Department of Cell and Developmental Biology, Division of Biosciences, The Centre for Cell and Molecular Dynamics, University College London, London, UK

**Keywords:** Biological techniques, Cell biology

## Abstract

Super-resolution (SR) 3D rendering allows superior quantitative analysis of intracellular structures but has largely been limited to fixed or ex vivo samples. Here we developed a method to perform SR live imaging of mitochondria during post-embryonic development of *C*. *elegans* larvae. Our workflow includes the drug-free mechanical immobilisation of animals using polystyrene nanobeads, which has previously not been used for in vivo SR imaging. Based on the alignment of moving objects and global threshold-based image segmentation, our method enables an efficient 3D reconstruction of individual mitochondria. We demonstrate for the first time that the frequency distribution of fluorescence intensities is not affected by photobleaching, and that global thresholding alone enables the quantitative comparison of mitochondria along timeseries. Our composite approach significantly improves the study of biological structures and processes in SR during *C. elegans* post-embryonic development. Furthermore, the discovery that image segmentation does not require any prior correction against photobleaching, a fundamental problem in fluorescence microscopy, will impact experimental strategies aimed at quantitatively studying the dynamics of organelles and other intracellular compartments in any biological system.

## Introduction

Mitochondria influence many aspects of eukaryotic cell physiology and energy metabolism^1,2^. For example, they play pivotal roles as signalling centres and participate in the regulation of cell survival and cell death^3–5^. Mitochondrial dysfunction due to defects in dynamics (fission–fusion cycles), biogenesis and/or trafficking has been linked to numerous human diseases, including neurodegenerative and cardiovascular disorders, diabetes, and cancer^6–11^. Therefore, intense efforts have been made to improve the analysis of these organelles on both the spatial and temporal scale. Electron microscopy (EM) has very high spatial resolution and allows, for example, the reconstruction of mitochondrial morphology and cristae organisation, as well as accurate quantification of these attributes^12,13^. However, EM requires fixed samples and thus cannot capture mitochondrial dynamics. Fluorescence microscopy of living samples permits direct observation of mitochondrial dynamics, and recent innovations in methodology have made it possible to combine this with high spatial resolution. Quantification of organelles by light microscopy is generally based on fluorescence intensity measurements or measurements of areas (2D) or volumes (3D) in binarized (=segmented) images. In the first case, the fluorescence intensity is usually measured within regions of interest (ROIs) and cannot be used to study organelle morphology. In the second case, segmentation of organelles is advantageous, because it also allows several additional analyses given that the size and morphology of objects are measurable. Numerous efforts have been made to develop methods that allow quantitative and morphological analyses of mitochondria and of other organelles from fluorescence microscopy images^14–22^. Thicker samples, like cell tissues or embryos, cannot be simplified as two-dimensional systems, and for this reason 3D reconstruction is critical^18^. However, the majority of methods reported to date for 3D reconstructions have focused on improving single z-stack (3D image) measurements and/or rendering mitochondria to track their movements. Monitoring mitochondrial quantity over time has been hampered by photobleaching, i.e., the irreversible loss of fluorescence due to photo-induced chemical alteration of fluorophores^23^. Multiple methodologies to correct for photobleaching have been proposed^24–27^, but they are often based on complex algorithms and/or assumptions that may introduce artefacts. Modelling photobleaching is further complicated by its spatial heterogeneity along the *Z*-axis during confocal microscopy^28–30^.

In this study, we have addressed these limitations using the model *Caenorhabditis elegans*. We have developed a complete composite approach that enables in vivo super-resolution (SR) live imaging and 3D rendering of mitochondria using reporter genes. Our approach also addresses and solves photobleaching. This time-series acquisition and analysis pipeline refines an existing nanobeads-based method to mechanically immobilise *C*. *elegans* L1 larvae^31^ and corrects for residual movements of mitochondrial objects (e.g., due to peristaltic movement of L1 larvae). This in turn allows for image deconvolution and subsequent 3D rendering. Finally, we show that global threshold-based binarization of images, before image segmentation, is not affected by moderate photobleaching during time-lapse fluorescence microscopy. Due to this property of global thresholding, we were able to monitor mitochondrial quantity during cell division. Taken together, the effective management of photobleaching and animal movements makes our composite methodology effective to quantitatively study the dynamics of mitochondria in developing *C*. *elegans* larvae.

## Results

### Mechanical immobilisation of *C*. *elegans* larvae enables in vivo imaging of mitochondria

**Fig. 1:**
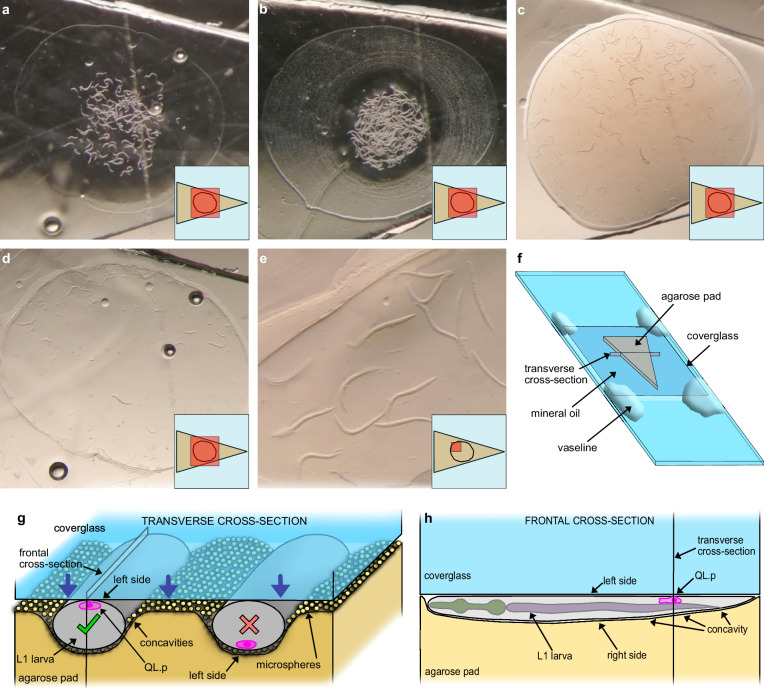
Mechanical immobilisation of L1 larvae. **a** Synchronised 4.5-h-old L1 larvae right after they were added with MPEG on a triangular agarose pad. **b** L1 larvae at the end of MPEG evaporation (concentric rings represent precipitated salts). **c** L1 larvae after the addition and partial evaporation of polystyrene microspheres. **d** L1 larvae after positioning the coverglass (the ring around larvae consists of precipitated microspheres and surfactant). **e** Detailed view of L1 larvae immobilised under the coverglass. The panel shows the ideal condition with almost no contact between animals. **f** Cartoon of the mount. The triangular pad is surrounded by mineral oil and surmounted by a 1½ coverglass. Vaseline is added only at the four corners of the coverglass. **g** Transverse cross-section of the mount. The coverglass presses L1 larvae against the agarose pad, causing mild compression of specimens (elliptic cross-section, left-right diameter is 20 μm or so) and the formation of concavities at the L1 cuticle-agar pad interface (visible also in (**e**)). In this condition, the microspheres between the L1 cuticle and the agar pad generate strong friction. Only the animal on the left faces the coverglass with its left side, QL.p is very close to the coverglass, and its division can be recorded in super-resolution. **h** Frontal cross-section of an immobilised L1 larva. The high content in agarose makes the pad deform mildly and allows perfect adhesion of the worm cuticle along the entire anterior-posterior axes. Microspheres are omitted to simplify the cartoon. Only (**a**, **d**) images are from the same replicate. **a**–**e** The small inserts at the lower right-hand side corners show the field-of-view (red boxes) relative to the triangular pad. Air bubbles in (**a**, **d**) are within the agarose pad and do not cause discontinuity to the surface.

**Fig. 2:**
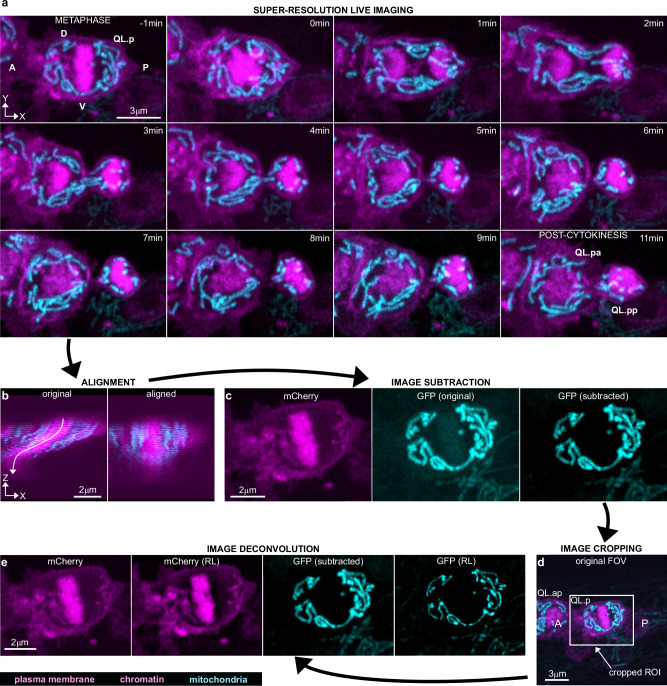
Super-resolution live imaging of QL.p division and downstream image processing. **a** Super-resolution live two-colour timeseries of QL.p division in animals expressing the transgenic multicopy array *bcIs153*. Plasma membrane (myristoylated mCherry) and chromatin (mCherry::his-24) are shown in magenta, and mitochondria (mtGFP) in cyan. Images are maximum intensity projections of aligned z-stacks (top view, *xy*). Image orientation has been changed for time-series illustration, whereas the original orientation can be seen in image processing (**c**–**e**). A anterior side, P posterior side, V ventral side, D dorsal side. **b** Lateral view (*xz*) of a z-stack showing QL.p at metaphase before (left) and after (right) alignment. Images refer to a cropped volume from the original z-stack (see also (**d**)). **c** Magenta z-stacks (plasma membrane + chromatin (mCherry), left) are subtracted from cyan z-stacks (‘GFP (original)’, centre). This arithmetic operation has minor effects on mtGFP signal distribution (‘GFP (subtracted)’) (see also Supplementary Fig. S3a). The subtraction is conducted on the entire z-stack. **d** Maximum intensity projection of an aligned and subtracted z-stack, which shows QL.ap in contact with the anterior side of QL.p at metaphase (see also Supplementary Fig. S2). The image represents the field-of-view (FOV) used to record QL.p division. The white box represents the ROI used to duplicate a new z-stack, after alignment, which will be used for the last step of image processing (**e**). **e** Richardson–Lucy (RL) deconvolution (step 4) of both magenta and cyan z-stacks. Deconvolved images are used for 3D rendering of QL.p cell shapes and mitochondria. Steps in (**b**–**e**) are performed in Fiji and describe the entire image processing workflow. **c**–**e** Refer to (**a**) at time −1 min.

We sought to quantitatively study the dynamics of mitochondria during the division of the QL.p neuroblast in *C*. *elegans* L1 larvae^32,33^. To perform SR live imaging, we optimised a protocol in which polystyrene nanobeads^31^ are used to immobilise L1 larvae that are pipetted onto a 10% agarose pad and overlaid with a coverglass (see 'Methods') (Fig. 1a–f). This immobilisation strategy proves to be effective for time-lapse imaging of QL.p in SR (Fig. 2a). However, SR resolution can only be achieved when animals contact the coverglass with their left side, where QL.p is located (Fig. 1g, h). The pressure that immobilised animals experience is not strong enough to arrest their development. (Fig. 2a and Supplementary Fig. S1a, ‘pipetting’). However, we did observe QL.p divisions with abnormal metaphase plates and abnormal mitochondrial morphologies (e.g., circular mitochondria) when animals were transferred to agarose pads with a worm pick instead of by pipetting (Supplementary Fig. S1a, ‘picking’). Circular mitochondria have already been reported in *C*. *elegans* and indicate stress and impaired activity of the electron transport chain (ETC)^34,35^. These observations indicate that our protocol minimises mechanical and other types of stress that animals may experience during mounting and immobilisation.

We also investigated how long animals can be studied after mounting by comparing morphologies of mitochondria and the duration of QL.p division (metaphase-to-post-cytokinesis interval) at different times after mounting (Supplementary Fig. S1b–f). Our measurements did not show any significant changes during imaging; however, we did observe an apparent decrease in the mean mitochondrial volume accompanied by an increase in both mitochondrial sphericity and number, starting ~1.5 h after mounting (70–100 min after immobilisation) (Supplementary Fig. S1b–d). This could be due to hypoxic stress, which has been shown to induce mitochondrial fragmentation in *C*. *elegans*^36^. Therefore, we recommend biological processes should not be studied more than 1.5 h post-immobilisation.

### Super-resolution time-series acquisition of QL.p cell division achieves single-organelle resolution

To follow mitochondrial partitioning during QL.p division, we generated a transgenic strain containing an integrated extrachromosomal array, with transgenes that express markers for DNA (mCherry::TEV-S::his-24), the plasma membrane (myristoylated mCherry)^37^ and the mitochondrial matrix (mtGFP). QL.p is smaller in size (5–7 μm at metaphase) than other cell types in *C*. *elegans*, such as body wall muscle^38^ or germ cells^39^, in which mitochondria have diameters of 300–800 nm. Indeed, we found that segmentation of QL.p mitochondria failed following acquisition with laser scanning confocal microscopy because the diameter of QL.p mitochondria are close to the diffraction limit^40^ for wavelengths in the visible spectrum. For this reason, we performed live imaging using the confocal laser scanning microscope Zeiss LSM980 with Airyscan2 in Airyscan SR mode. This microscope was equipped with an oil-immersion objective (Zeiss C Plan-Apochromat 63×/1.4 Oil DIC M27) with a nominal resolution of 240 nm in *xy* and 720 nm in *z* in standard LSM mode and 120 nm in *xy* and 360 nm in *z* in Airyscan SR^41^. Based on images obtained with this system, we estimate the diameters of QL.p mitochondria to be between 100 and 300 nm. Despite immobilisation, animals still exhibited occasional muscle contractions, and these shifted the position of QL.p throughout both z-stack acquisition and the timeseries (Fig. 2b, ‘original’, Supplementary Videos 1 and 2). To follow QL.p division at a 1-min time resolution (Fig. 2a), we used a field-of-view (FOV) that is larger than the *xy* dimensions of QL.p (Supplementary Fig. 1 and Supplementary Video 2). In addition, we manually recentred the FOV on QL.p between acquisitions every time QL.p had moved out of the monitored area. Using this approach, we ensured that QL.p could always be found within the FOV over the entire timeseries. We noticed that a large FOV is more practical due to the relatively slow dual-colour acquisition speed of our imaging system in Airyscan SR mode (see 'Methods'). We found that we could obtain images with very high contrast and negligible motion blur by using higher laser power (see 'Methods') and short dwell times per pixel (1.03 µs). The z-stacks also include additional sections above and below QL.p to allow for cell movements along the *z*-axis as QL.p divides to generate QL.pa and QL.pp (Supplementary Fig. S2 and Supplementary Video 1). We also performed oversampling in *z* (sampling at a shorter z-step than recommended by ZenBlue software) to increase the information available to process z-stacks (see below). The integration of our immobilisation method and image acquisition strategy enabled us to successfully acquire timeseries of QL.p divisions at single-organelle resolution.

### Image processing enables the 3D rendering of mitochondria in moving QL.p cells

To quantitatively study the dynamics of mitochondria during QL.p division, we focused on two time points along the timeseries (Fig. 2a): the end of metaphase (last metaphase image in the timeseries, which is referred to as “metaphase”) and the end of cytokinesis (first image showing no mitochondria in the intercellular bridge during late cytokinesis, which is referred to as “post-cytokinesis”). Dual-colour, Airyscan-processed z-stacks of metaphase and post-cytokinesis are imported into Fiji, and image processing is performed in four steps: (i) z-stack alignment, (ii) image subtraction, (iii) image cropping, and (iv) deconvolution (image cropping can precede image subtraction). As our immobilisation protocol cannot avoid minimal movements of QL.p (or QL.p daughters) during z-stack acquisition (Supplementary Video 1), image alignment is needed to reconstitute the correct distribution of 3D information across z-stacks (Fig. 2b). Image alignment is possible only if cell and mitochondria shapes move in the *xy* plane without morphological distortions during z-stack acquisition. Timeseries that do not fulfil these criteria are discarded. (Less than 5% of the timeseries had to be discarded and discarded timeseries were mainly of animals that exhibited excessive levels of movements such as lateral head and/or tail movements.) Next, mCherry z-stacks (100% of the mCherry signal in the red channel) are subtracted from mtGFP z-stacks (Fig. 2c). This subtraction is necessary because the GFP-exciting 488 nm wavelength also excites mCherry (by 8%)^42^, thereby causing mCherry fluorescence to bleed into mtGFP images (Fig. 2c, ‘GFP (original)’). This subtraction did not cause the removal of mitochondria from images because the mtGFP signal was brighter than the mCherry signal in the transgenic strain (see 'Methods' for more details) (Supplementary Figs. S3a and S4a, b). To verify that this subtraction did not produce artefacts (i.e. removal of mitochondria), we subtracted 12% rather than 100% of the mCherry signal from mtGFP images (Supplementary Fig. S4c). Both subtractions, using either 100% or 12% of the mCherry signal, produced similar images in terms of contrast enhancement, although those generated by 12% mCherry signal subtraction appear more similar to the unprocessed images (Supplementary Fig. S4b, c—“top view (isolines)” plots). For all subsequent analyses, we used images that were processed through 100% mCherry signal subtraction.

**Fig. 3:**
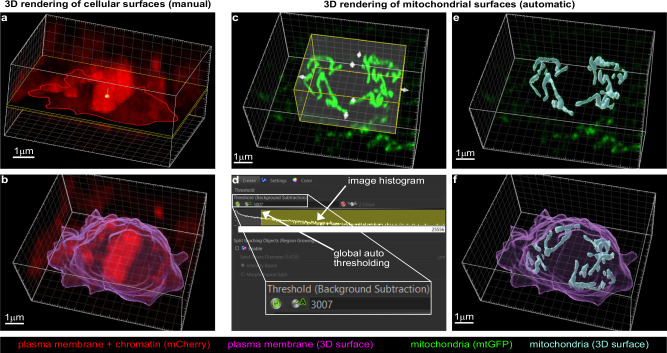
3D rendering of QL.p plasma membrane and mitochondria in Imaris. **a** Manual 3D rendering (Surface algorithm) of QL.p plasma membrane. ROIs (red contours following myristoylated mCherry distributions) are drawn manually on every slice of processed z-stacks. **b** 3D model of QL.p plasma membrane at metaphase (magenta). **c** Automatic 3D rendering (Surface algorithm) of QL.p mitochondria (mtGFP). 3D regions of interest (yellow) are enabled and manually defined to determine volumes in which 3D surfaces of mitochondria are computed. **d** 3D surfaces of mitochondria are rendered through automatic (A) global thresholds and local background subtraction. Global thresholds define the number of voxels that compose mitochondria (in the panel, yellow selection in the log-scaled grey value frequency distribution between 3007 (threshold value) and 25,556 (max value). **e** 3D surfaces of QL.p mitochondria at metaphase (cyan). **f** Overview of QL.p cell and mitochondria 3D models. **a**–**f** The white box represents the dimensions of the z-stack previously processed in Fiji (see also Fig. 2d, e).

Next, a smaller z-stack that includes mostly the cell of interest is cropped from the larger aligned image. By doing so, z-stacks are smaller for metaphase than for post-cytokinesis since the QL.p is rounder at metaphase and occupies a smaller surface in the *xy* plane (Fig. 2d). Cropping is useful to reduce the image size (i.e., faster deconvolution) and to avoid visual artefacts that are generated during deconvolution that are specific to mCherry (Supplementary Fig. S3d). These visual artefacts are caused by translations and rotations of z-stack slices during alignment that result in high contrast due to the absence of pixel information (grey value = 0) at the edges of the aligned z-stacks. The same artefacts are not visible in the GFP channel because all background grey values are either 0 or close to 0 after subtraction, meaning there is no contrast between the edges of the image and pixel-free portions of the image. Finally, mCherry and mtGFP z-stacks are deconvolved through the Richardson–Lucy (RL) algorithm (Fig. 2e) using theoretical Point Spread Functions (PSFs) specific to mCherry and mtGFP, respectively (Supplementary Fig. S5g). The computation of PSFs is simplified by excluding the contribution of positive longitudinal spherical aberration, which is present due to the use of a 63× 1.4 NA oil-immersion objective. We developed a simple methodology based on trigonometry to calculate the longitudinal shift of QL.p sections (Supplementary Fig. S5a–f). We determined that on average the longitudinal shift is 290 nm (less than 1/10 of the apparent QL.p thickness), with larger (~500 nm) and smaller (~80 nm) longitudinal shifts further away and closer, respectively, from the coverglass (Supplementary Figs. S2 and S5e, f). By simplifying QL.p as an ellipse, and using the average longitudinal shift to calculate the difference between apparent and real dimensions (thickness and volume) (Supplementary Fig. S5f), we estimated that QL.p appears to be 13% bigger than its actual size. This minor aberration causes the thickening of all objects in the image, with negligible alterations of their relative positions. Thus, it does not cause artificial fusion of mitochondria, because it results in outdistancing along the optical axis (Supplementary Fig. S5f). The small difference between the apparent and actual QL.p dimension is a consequence of the subcuticular position of QL.p, which minimises the effects of light refraction at the coverglass-cuticle interface (Supplementary Fig. S5b). After deconvolution, images are merged and opened in Imaris 9.8 for 3D rendering (Surface algorithm). Cell volumes are constructed by manually drawing ROIs on each section (Fig. 3a). Mitochondria are automatically constructed within cropped 3D ROIs (Fig. 3c), which excludes most mitochondria belonging to neighbouring cells. The remaining mitochondria are then manually curated, leaving only mitochondria belonging to the cell of interest. Within the cropped ROIs, global autothresholding determines the appropriate fluorescence intensity cut-off to segment mitochondrial volumes (Fig. 3d). Finally, we 3D-render cell and mitochondrial volumes (Fig. 3f and Supplementary Fig. S5d), and these can then be used to analyse quantities, morphologies, and distribution before and after cell division.

### Photobleaching affects fluorescence intensities, but not segmentation of mitochondria in Imaris

**Fig. 4:**
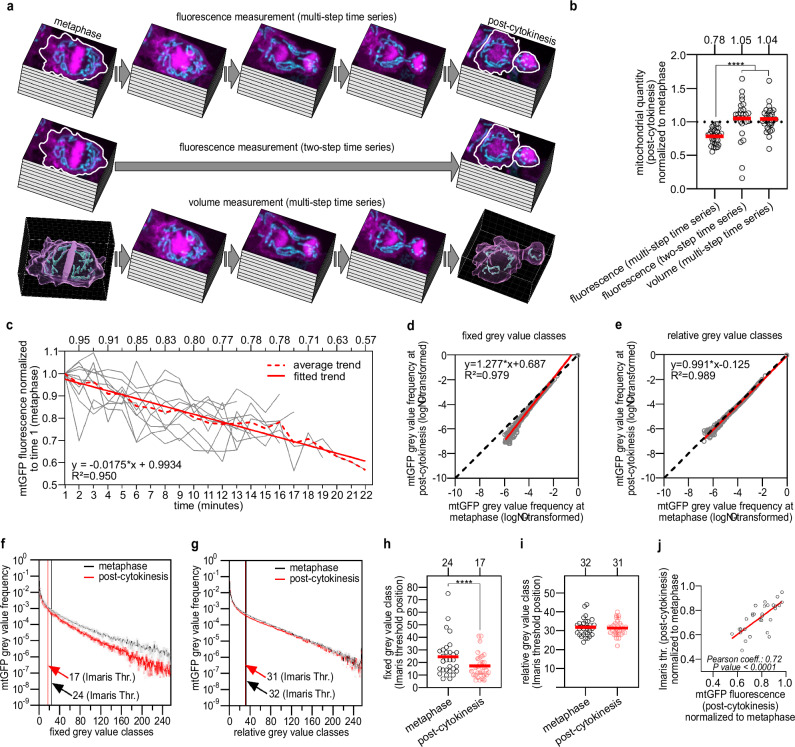
Photobleaching does not interfere with threshold-based segmentation of mitochondria in Imaris using mtGFP. **a** Schematic of mitochondrial quantity measurement in animals expressing *bcIs153*. mtGFP (mean Integrated Density, Fiji) measurement within cell contours (white ROIs) at both metaphase and post-cytokinesis, either along multi-step (top) or two-step (centre) timeseries. Bottom: mitochondrial volume measurement (Imaris) at both metaphase and post-cytokinesis along multi-step timeseries. Quantifications were performed on z-stacks after image processing (fluorescence) or after 3D rendering (volume). **b** Mitochondrial quantities (post-cytokinesis) normalised to respective quantities at metaphase (*n* = 30, 24, 30). **c** Average mtGFP photobleaching from trends constructed on fluorescence values normalised to time 1 (*n* = 10, referring to panel **a**, top). **d**, **e** Linear regression modelling the relationship (see 'Methods') between log-transformed average grey value frequencies (*n* = 30) measured on 256 fixed or relative grey value classes both at metaphase and post-cytokinesis from processed images (see also Supplementary Fig. S9). **f**, **g** Average mtGFP grey value frequency distributions generated on 256 fixed or relative grey value classes from processed images (*n* = 30) (see also Supplementary Fig. S9) (error bars = SEM). Mean grey value classes to which Imaris Surface threshold values belong are shown. **h**, **i** Individual fixed or relative grey value classes to which Imaris Surface threshold values belong (*n* = 30). **j** Correlation between normalised Imaris Surface threshold values and normalised mtGFP fluorescence (photobleaching) at post-cytokinesis. *P* values are calculated using a Kruskal–Wallis non-parametric rank test with Benjamini, Krieger and Yekutieli correction (**b**), a Wilcoxon matched-pairs signed rank test (**h**) or a paired *t* test (**i**). Normality was tested with the Shapiro–Wilk test. *P value ≤ 0.05; **P value ≤ 0.01; ***P value ≤ 0.001; ****P value ≤ 0.0001. **c** We used aligned and subtracted z-stacks, while in (**d**–**j**) we used fully processed z-stacks. Red bars = mean (**b**, **h**, **i**).

Next, we investigated whether our method allows the direct comparison between mitochondrial quantity in mother and daughter cells. To that end, we assessed photobleaching and how it affects the estimation of mitochondrial volume. The fluorescence decay of mtGFP along multi-step timeseries starting at metaphase and concluding at post-cytokinesis averaged 22% (average normalised fluorescence intensity: 0.78 ± 0.12 sd) in processed images (Fig. 4b), which is significant (one sample *t* test against the hypothetical 1.0 value, *P* value < 0.0001). The mtGFP fluorescence decay is linear with an average decay rate of 1.75% per minute (slope = −0.0175, 95% confidence interval = −0.0194 to −0.0157) (Fig. 4c). The decay of mtGFP fluorescence in all timeseries is characterised by fluctuations, which can be as large as 20% between consecutive time points (Supplementary Fig. S6). We did not observe a significant change in fluorescence when taking only two z-stacks, one at metaphase and one at post-cytokinesis (two-step timeseries) (average normalised fluorescence intensity: 1.049 ± 0.314 sd) (one sample Wilcoxon test, median = 1.11, *P* value = 0.09) (Fig. 4b). This strongly suggests that mitochondrial quantity is invariant (no biogenesis or mitophagy) during QL.p division (between metaphase and post-cytokinesis). Strikingly, we observed that mitochondrial volume 3D rendered from multi-step time-series images (the same multi-step timeseries analysed above for measuring fluorescence intensities) are also invariant (average normalised volume: 1.040 ± 0.182 sd) (one sample *t* test, *P* value = 0.20) (Fig. 4b). Of note, we were able to reproduce these results using images that were processed through 12% mCherry signal subtraction (Supplementary Fig. S7).

To exclude that these results are specific to the use of GFP and/or targeting fluorescent proteins to the mitochondrial matrix, we used a different mitochondrial marker. To label the cytosolic side of the outer mitochondrial membrane, we generated a transgene that mediates the expression of a mKate2 fusion protein with the N-terminal region (residues 1–54) of *C. elegans* TOMM-20^43^, which includes the mitochondrial targeting signal and the transmembrane domain, fused to its N-terminus (TOMM-20(N)-mKate2) (Supplementary Fig. S8a, b). The TOMM-20(N)-mKate2 fluorescence decay is linear with an average decay rate of 4.13% per minute (slope = −0.0310 sampling every 45 s, 95% confidence interval = −0.03648 to −0.02552) (Supplementary Fig. S8c). The decay of TOMM-20(N)-mKate2 fluorescence in all timeseries is also characterised by fluctuations, which can be as large as 30% between consecutive time points. The fluorescence decay of TOMM-20(N)-mKate2 along multi-step timeseries starting at metaphase and concluding at post-cytokinesis averaged 31% (average normalised fluorescence intensity: 0.69 ± 0.10 sd), which is significant (one sample Wilcoxon test, median = 0.69, *P* value < 0.0001) (Supplementary Fig. S8d). We did not observe a significant change in fluorescence when we took two z-stacks, one at metaphase and one post-cytokinesis (two-step timeseries) (average normalised fluorescence intensity: 0.970 ± 0.164 sd) (one sample Wilcoxon test, median = 0.93, *P* value = 0.07), or when we segmented mitochondria in 3D (Imaris) using multi-step timeseries (average normalised volume: 1.024 ± 0.263 sd) (one sample Wilcoxon test, median = 1.00, *P* value = 0.93) (Supplementary Fig. S8b). Therefore, we find that accurate segmentation of mitochondria in Imaris does not require correction for photobleaching, regardless of the fluorophore used and the mitochondrial compartment labelled.

### Photobleaching affects the minimum-maximum range of image histograms

Segmentation in Imaris Surface is based on global thresholding, which binarizes images by analysing the grey value histograms of z-stacks (referred to as 'image histograms'), which were cropped during 3D rendering of mitochondria (Fig. 3c, box highlighted in yellow). We investigated how image histograms change during time-lapse imaging and why photobleaching did not affect segmentation in Imaris. We generated two different types of image histograms for both metaphase and post-cytokinesis processed images: 'fixed' histograms, where the minimum-maximum (min–max) intensity range is invariant, and 'relative' histograms (see the next section), where the grey value min–max intensity range is specific to every individual z-stack (Supplementary Fig. S9a, b). We first fitted a linear regression between log-transformed mean frequencies from metaphase and post-cytokinesis fixed histograms (Fig. 4d). The regression slope (1.277) was significantly higher than the hypothetical 1.0 value (*y* = *x*) (*F*_1,248_ = 532.3, *P* value < 0.0001), indicating that fluorescence intensities are reduced at post-cytokinesis (i.e., mtGFP was photobleached) compared to metaphase. This reduction in intensities suggests that fixed image histograms differ between metaphase and post-cytokinesis. Indeed, image histograms at post-cytokinesis are left-shifted compared to metaphase (Fig. 4f and Supplementary Fig. S9a, right), which means that photobleaching lowers grey value frequencies. As a result, the fixed min–max interval is narrowed along the *x* axis (Fig. 4f and Supplementary Fig. S9a, right). To test and validate these differences, we fitted non-linear functions to the individual grey value frequency distributions of images at metaphase and post-cytokinesis. Integrating under the respective curves and taking the difference in area under the curves between metaphase and post-cytokinesis, we approximated and tested the effects of photobleaching for each division in a pairwise manner (see Supplementary Method: Figs. S15–S20). We observed a significant difference between metaphase and post-cytokinesis median δ area of 512.4 (±756.5 sd), which accounts for a difference of −15% in area (one sample Wilcoxon test, *P* value < 0.0001) (Supplementary Fig. S10a). We also examined the position of threshold values (Imaris Surface) along fixed grey value frequency distributions by assigning a class to each value (Fig. 4f). With this approach, we noticed that the positions of threshold values are significantly reduced at post-cytokinesis (Fig. 4h).

To determine whether these results are an artifact of our image processing protocol, we repeated the same analysis on the original FOV of unprocessed images (i.e., images were not subjected to any transformation) (Supplementary Fig. S10e). By fitting a linear regression between log-transformed mean frequencies of metaphase and post-cytokinesis fixed grey value frequency distribution, we observed a similar difference between the regression slope (1.216) and the hypothetical 1.0 slope (*F*_1,241_ = 334.1, *P* value < 0.0001) (Supplementary Fig. S10c, left). We also observed the same left shift of the grey value frequency distribution at post-cytokinesis (Supplementary Fig. S10d, left), which is reflected by an average δ area difference between metaphase and post-cytokinesis of 150.9 ( ± 96.5 sd) (−5% area difference) (one sample *t* test, *P* value <0.0001) (Supplementary Fig. S10b and Supplementary Method: Figs. S21–S23). As a result, the position of threshold (for the selection of Isodata algorithm, see below) values is significantly reduced at post-cytokinesis (Supplementary Fig. S10d, left, S10f), as observed in processed images. Thus, results on unprocessed images corroborate the observations made on processed images. In conclusion, based on our analysis on fixed histograms, Imaris surface thresholding maintains accuracy despite the change in the min–max mtGFP intensity range caused by photobleaching.

### Photobleaching has negligible effects on the grey value frequency distribution of image histograms

The analysis on fixed histograms revealed that photobleaching affects min–max ranges and the position of thresholds. However, changes in the ranges themselves do not provide information about how the frequency distribution changes between the minimum and maximum values over time. Therefore, we focused on metaphase and post-cytokinesis relative histograms, the min–max range of which is image-specific. By log-transforming their mean frequencies and fitting a linear regression as done in the previous section for fixed histograms, we observed a minimal difference: the slope (0.991) does not differ in a statistically significant manner from 1.0 (*F*_1254_ = 1.948, *P* value = 0.16) (Fig. 4e). This suggests that relative frequency distributions at metaphase and post-cytokinesis are similar (Fig. 4g and Supplementary Fig. S9a, left). Similarly fitting non-linear functions to the frequency distributions and integrating under the curves (see Supplementary Method: Figs. S15–S20), we noticed a shrinking of the δ area between the curves compared to fixed thresholding. We observed a significant average difference in δ area between metaphase and post-cytokinesis of 47.4 (± 108.1), which accounts for only a −2% area difference (one sample *t* test, *P* value = 0.023) (Supplementary Fig. S10a). The position of Imaris surface thresholds (the same thresholds as mentioned above for the fixed histogram analysis) along relative histograms does not change over time (Fig. 4i).

Again, to determine whether these results are an artifact of our image processing protocol, we repeated the same analysis on unprocessed images (i.e., images were not subjected to any transformation) (Supplementary Fig. S10e). By fitting a linear regression between log-transformed mean frequencies of metaphase and post-cytokinesis relative histograms, we observed a small but statistically significant difference between the regression slope (0.994) and the hypothetical 1.0 slope (*F*_1254_ = 4.348, *P* value = 0.0381) (Supplementary Fig. S10c, right). Relative thresholding made the frequency distributions at metaphase and post-cytokinesis align (Fig. S10d, right). This is also reflected by the average δ area between curves dropping to −34.8 ( ± 78.7 sd), constituting a + 1% difference in area between metaphase and post-cytokinesis curves (one sample *t* test, *P* value = 0.11) (Supplementary Fig. S10b and Supplementary Method: Figs. S21–S23). As a result of this small difference, the position of threshold values does not change during photobleaching (Supplementary Fig. S10g). These results corroborate the observations made on processed images. These results strongly suggest that Imaris surface thresholding does not compensate for photobleaching, but that photobleaching has minor effects on the frequency distribution of image histograms. This indicates that changing the min–max intensity ranges does not affect the frequency distributions and that Imaris surface thresholding is not based on the min–max ranges, but on the grey value frequency distribution of image histograms. As a result, during mtGFP photobleaching, the position of the threshold values is shifted to the left as a direct result of the left shift undergone by the frequency distributions. However, since both frequency distributions and threshold positions are left-shifted, the threshold positions do not change relative to the frequency distribution. Therefore, the left shift of threshold values is positively correlated with the extent of photobleaching (Fig. 4j and Supplementary Fig. S10h). In conclusion, photobleaching has negligible effects on grey value frequency distributions during segmentation in Imaris Surface.

### Photobleaching does not affect global threshold-based segmentation

Segmentation in Imaris enables the comparison between mitochondrial quantities along timeseries. To test whether the relationship between global thresholds and gray value frequency distribution is specific to Imaris Surface or is a general phenomenon, we developed a protocol for 2D-rendering of mitochondria in Fiji (ImageJ 1.53f51). This protocol reproduces in Fiji the main steps followed in Imaris Surface. We compared most threshold algorithms available in Fiji with that of Imaris Surface (Supplementary Fig. S11a, see 'Methods'). To simplify the measurements of mitochondrial quantity changes during QL.p division, we did not remove objects from neighbouring cells that were identified during image binarization (either in Fiji or in Imaris Surface) (Supplementary Fig. S11a, orange arrows). Next, we reused the same threshold values achieved with Imaris Surface in Fiji (*“Fiji (Imaris Surface)”*) as a control for comparison with the other algorithms (Supplementary Fig. S11b) and compared normalised mitochondrial quantities at post-cytokinesis. Using this approach, we found that the normalised mitochondrial quantities generated by the algorithms in Fiji are not significantly different from those generated by Imaris Surface (Supplementary Fig. S11b). Normalised quantities were greater than 1 in this analysis due to the larger 3D ROIs used to render post-cytokinesis mitochondria (Fig. 3c and Supplementary Fig. S3c). Larger 3D ROIs increase the likelihood of including objects from neighbouring cells in the 3D rendering, which artificially inflates QL.p mitochondrial quantities compared to metaphase. The similarity between all thresholding algorithms (Supplementary Fig. S11b) suggests that the resiliency observed in Imaris Surface during photobleaching is not specific to Imaris but is a shared property of many global thresholding algorithms.

We tested whether the mitochondrial quantity invariance that we observed using Imaris Surface is reproducible in Fiji. To this end, we constructed a protocol to segment mitochondria in Fiji that reproduces in 2D all steps followed in Imaris Surface, apart from local background subtraction (Fig. 3d) and smoothing (see 'Methods'). For this protocol, we select a global thresholding algorithm in Fiji showing a similar performance to Imaris Surface. We compared cumulative mitochondrial quantities (in pixels) in QL.p at metaphase between the Fiji threshold algorithms and Imaris Surface (“Fiji (Imaris Surface)”) (Supplementary Fig. S11c). The significant difference between mitochondrial quantities in Imaris (“Imaris Surface (3D)”) and Fiji (“Fiji (Imaris Surface)”) is probably because in Fiji, we did not reproduce the local background subtraction and smoothing during thresholding (Supplementary Fig. S11c and Fig. 3d, see 'Methods'). We found that only the Default and Isodata algorithms generate QL.p mitochondrial quantities that are not significantly different to Imaris Surface. Since Default is a variation of Isodata^44^, we used Isodata^45,46^ in our protocol. Next, from the same z-stacks processed in Fiji and used in Imaris (Supplementary Fig. S12a–c), we duplicated 3D ROIs corresponding to those manually determined in Imaris for 3D rendering of mitochondria and binarized them with Isodata (Supplementary Fig. S12d and Fig. 3c). We then drew ROIs along the plasma membrane of QL.p, or QL.p’s daughter cells, to remove any object localised outside of the cell boundaries (Supplementary Fig. S12e, f, see 'Methods'). Finally, new ROIs were generated on the remaining mitochondrial objects for measurements (Supplementary Fig. S12g). As observed in Imaris Surface (Fig. 4g), the position of Isodata thresholds does not change significantly between the relative image histograms at metaphase and post-cytokinesis (Supplementary Fig. S13a, b). However, their positions are significantly different from those produced by Imaris Surface (Supplementary Fig. S13c, d). This suggests that using Fiji Isodata, global threshold positions are also left-shifted during photobleaching, such that they do not change relative to the grey value frequency distributions as photobleaching occurs. As a result, Isodata thresholds at post-cytokinesis, normalised to metaphase, are strongly correlated with the decay of mtGFP brightness (Supplementary Fig. S13e), as we observed for Imaris Surface (Fig. 4j). Normalised Imaris Surface and Isodata thresholds are also well correlated, which is not surprising since they are both variants of the same algorithm^46^ (Supplementary Fig. S13f). Finally, as observed with 3D rendering in Imaris (Supplementary Fig. S13g), mitochondrial quantities do not change during photobleaching when generated through 2D-rendering in Fiji (One sample *t* test, *P* value = 0.12). Overall, the reproducibility of the mitochondrial quantity invariance during photobleaching in Fiji corroborates the results obtained in Imaris Surface and confirms that the frequency distribution in image histograms is minimally affected by photobleaching. Importantly, the observation that the different algorithms that we tested performed similarly suggests that the resiliency of grey value frequency distributions during photobleaching is generalisable. Thus, thresholding-based segmentation of mitochondria (as well as other organelles or intracellular compartments) allows the quantitative comparison over timeseries irrespective of photobleaching, image analysis software and thresholding algorithm.

## Discussion

Linking together various aspects of mitochondrial biology, such as quantity, morphology, position, and trafficking in the same experiment has fundamental implications and can uncover how these organelles impact development, tissue homoeostasis, energy homoeostasis, and pathology. Although advances have been made in fluorescence microscopy, image processing and segmentation, photobleaching remains problematic and limits the analysis of mitochondria over short to mid timescales. In this study, we have addressed several problems in the analysis of mitochondria in vivo, such as: the drug-free immobilisation of *C. elegans* larvae, the 3D reconstruction of mitochondria in non-static cells, and the impact of photobleaching. We integrated readily available tools into a method that allows the tracking in SR of the temporal dynamics of mitochondria during a developmental process. We optimised an existing method to achieve effective, but gentle and drug-free, mechanical immobilisation of *C. elegans* larvae for short-term confocal microscopy (up to 1.5 h). Although our immobilisation method is unsuitable for long-term imaging, it is easy and quick to perform and allows the acquisition of up to 30 timeseries, one-by-one in a single day. Our immobilisation protocol is inexpensive, versatile, and suitable for various biological contexts and does not require specialised equipment. We are confident that older developmental stages of *C. elegans* can also be mechanically immobilised following our protocol by adjusting the xy sizes of the coverglass and the agarose pad along with the diameter of the nanobeads. However, for older *C. elegans* stages, SR can be achieved only if the imaged objects are relatively close to the coverglass interface as the light scattering through thicker animals inevitably increases the amount of blur. Our methodology can also be applied to imaging entire L1 or L2 stage animals, using a SoRA spinning-disk confocal microscope^47,48^, at image resolutions similar to that achieved in this study. In larger cells with mitochondria of diameters larger than 300 nm, such as body wall muscle or germ cells^38,39^, the image resolution that we achieved here may be sufficient to study even mitochondrial ultrastructures, such as cristae. To increase image resolution even further, nanoscopy techniques like STED^49,50^ may be necessary, which requires high laser power and prolonged irradiation. Alternatively, structured-illumination microscopy (SIM) systems, which achieve a nominal resolution of 60 nm in *xy*, could be used. One such example is the Zeiss Elyra 7 microscope, implemented with SIM^2^ image reconstruction. We imaged QL.p mitochondria using this system and observed that the slower lattice imaging mode can cause extensive motion blur in animals immobilised with the method presented here (Supplementary Fig. S14a, b). On the contrary, the faster apotome acquisition produced images without motion blur (Supplementary Fig. S14c, d). Even though apotome images have an intrinsic lower resolution compared to lattice images, the SIM^2^ reconstruction process restored image resolution to similar levels that are typical of lattice imaging (Supplementary Fig. S14a, d). Moreover, by imaging small FOVs and reducing the exposure time, we were able to acquire z-stacks of QL.p, in just 1–2 s, thereby avoiding motion blur completely. Therefore, our immobilisation method can also be used with fast nanoscopy systems to reach image resolution below 100 nm.

Even though laser scanning confocal microscopy is limited by slower image acquisition compared with spinning-disk confocal microscopy^51^, the immobilisation we achieved enabled us to crop small FOVs and to increase the speed of image acquisition. The implementation of image alignment in our processing pipeline solved the problem of cell movements during acquisition and improved deconvolution and 3D rendering of imaged non-static objects. In the case of fluorescence bleedthrough, as we describe here, we recommend investigating the relative brightness of the different fluorescent proteins to avoid artefactually removing information from the data. Moreover, even if both lasers are set at the same power, we recommend measuring laser intensities for different wavelengths with a photometer to better estimate how much signal should be subtracted. Analysing the mtGFP fluorescence decay during the acquisition of multi-step timeseries, we observed fluctuations in fluorescence intensity between consecutive z-stack acquisitions. These fluctuations may have been due to transient changes in intracellular pH^52^ and/or redox status^53^ during QL.p division. Unlike wtGFP (also known as avGFP^54^), the GFP variant used to generate mtGFP in this study (GFP(S65C)) may be more sensitive to acidic conditions, as shown for the spectrally similar GFP(S65T)^55^ and EGFP (GFP(F64L/S65T))^56^. In that case, GFP(S65C) photochemical changes would be expected to occur mostly below pH7 as shown for GFP(S65T) and EGFP; however, in active mitochondria, the matrix pH is generally 7.5–8.2^57^. Although we have evidence that mitochondria are polarised (electrochemical gradient across the inner mitochondrial membrane) in our imaging conditions, we cannot exclude that changes in cytosolic pH during QL.p division^52^ affect GFP(S65C) by impacting the mitochondrial matrix pH. In addition, GFP(S65C) may undergo reversible green-to-red photoconversion (redding) as shown for EGFP^58,59^ in environments with high levels of reducing agents, such as the mitochondrial matrix. Similarly, mKate2 signal intensity fluctuations may be caused by the presence of reducing agents or by a non-canonical type of red-to-green phototransformation, which has been described for both mKate and mKate2^60^.

Finally, we extensively studied image histograms and found that image segmentation, using global thresholding, is unaffected by photobleaching. This contradicts the findings of Moo et al.^61^, who observed an apparent decrease in cell volume over time during ex vivo confocal microscopy of cartilage chondrocytes. These authors used the Ridler–Calvard algorithm^46^ (from which IsoData and Imaris Surface thresholding were developed) and noticed a left shift in scan-specific threshold positions during time-series acquisitions^61^. They might have failed to observe cell volume invariance during timeseries due to: (1) Lack of correction for image blur. This would explain why their results regarding cell volume changes over time were inconsistent between Otzu, Ridler-Calvard and 40% maximum intensity algorithms^61^; (2) Sampling bias for high foreground-to-background contrast over the entire timeseries. Specifically, their arbitrary sampling bias may have masked the effectiveness of thresholding against photobleaching; (3) the technical limitations of their imaging system^61^. We observed invariant mitochondrial quantities in processed images using multiple algorithms in Fiji (Supplementary Fig. S11b) and using different image processing software. Moreover, these results were reproducible using unprocessed or processed images with different image subtractions. In addition, our results are not limited to using GFP(S65C) or to targeting this GFP variant to the mitochondrial matrix as we obtained the same result using mKate2 targeted to the cytosolic side of the outer mitochondrial membrane. This strongly suggests that our findings on photobleaching are not determined by the type of fluorescent protein and by the subcellular localisation. Our results are also consistent with a previous description, according to which global thresholding, as used here, is based on image grey-level histograms^62^. While global thresholding is based on image histograms, photobleaching affects the min–max grey value ranges, but not the image-specific (relative) profiles of image histograms. Therefore, the grey value min–max ranges of image histograms are reduced during photobleaching, while global thresholding consistently binarizes images in the same section of histograms due to the invariance in their image-specific (relative) profiles. Because of this, global thresholds change (=left shifting along the *x* axis of image histograms) proportionally to the extent of photobleaching (Fig. 4j and Supplementary Figs. S10h and S13e), which determines the invariance in the quantity (i.e., the number of pixels or voxels) of the segmented objects. Therefore, we propose that the dynamics of mitochondria (or other intracellular compartments) can be studied quantitatively in timeseries with global threshold-based segmentation requiring no prior correction for photobleaching. However, our 3D rendering methodology and new principles on image histograms may not be applicable to the smallest cellular structures, such as F-actin or microtubules (7 nm or 24 nm in diameter, respectively). To image those, different microscopy techniques, such as structured-illumination microscopy, and different segmentation methods may be needed^63^.

Alternative imaging systems that minimise photobleaching, such as multiphoton^64^, lattice light-sheet^65^ and csLFM microscopy^66^, are all characterised at most by the near-diffraction limit resolution. In contrast, laser scanning confocal microscopy (LSCM) systems, such as the super-resolution Zeiss LSM980 with Airyscan2, produce relatively high levels of photobleaching^67^. Nevertheless, concerning image analysis, we have largely demonstrated that photobleaching has minimal impact on image segmentation using super-resolution LSCM. Therefore, LSCM enables the study of quantitative and spatiotemporal changes of intracellular compartments, such as mitochondria, even when photobleaching is not avoidable, which represents an excellent compromise between photobleaching and the achievement of super-resolution.

In conclusion, our new methodology, which ignores photobleaching, provides new opportunities to explore aspects of cell biology that have remained unexplored due to imaging limitations. Moreover, we are confident that our findings on image histograms will contribute to the development of new image analysis tools that better address photobleaching.

## Methods (protocol)

### *C. elegans* strains

The *C. elegans* strain MD4688 was cultured on Nematode Growth Medium (NGM) in medium Petri dishes seeded with *E. coli* OP50. MD4688, used for all experiments except for the one relative to Supplementary Fig. S8 (strain MD4802), expresses the MiniSOG-integrated^68^ extrachromosomal array *bcIs153 IV* (pBC1590; *Ptoe-2::mtGFP::unc-54-3’UTR*) [this work] + *Pegl‐17::myristoilated-mCherry::pie-1-3’UTR* + *Pegl‐17::mCherry::TEV-S::his‐24::his-24-3’UTR* + PRF4(*rol-6(su1006dm)*). The transgenes Pegl‐17myristoilated-mCherry::pie-1-3’UTR and Pegl‐17mCherry::TEV-S::his‐24::his-24-3’UTR were amplified (Q5 PCR) from RDV55 (*rdvIs1*)^37^ genomic DNA using the primers CCTTCCGTTCTATGGAACACTC + AAAAAAAGTAAATTATTTTTGGCTTTTTTGG and CACTTCCGTTCTATGGAACACTC + GAAGACGTTGAACGTCAAATTAT, respectively. MD4802 expresses the MiniSOG-integrated extrachromosomal array *bcIs164* (unmapped) (pBC1974 (*Pegl-17::myristoilated-SFmTurquoise2ox::pie-1-3’UTR*) (this work) + pBC1973 (*Pegl-17::SFmTurquoise2ox-TEV-S::his-24::his-24-3’UTR*) [this work] + pBC1999 (*Ptoe-2::tomm-20(N-term)::mKate2::tbb-2-3’UTR*) [this work] + pBC1996 (*Ptoe-2::GFP::tbb-2::unc-54-3’UTR*) [this work] + PRF4(*rol-6(su1006dm)*). Worms were maintained at 20°C before synchronising L1 larvae.

### Cloning and plasmid construction

**pBC1973**: The plasmid pBC1858 was digested with ApaI and EcoRI to generate the vector backbone. The *egl-17* promoter insert was generated from pBC1858 template with oligos TGGATCCCCCGGGCTGCAGGAATTCGATcagatggatgtttactgccaactg and agctcacatttcgggcac. The SFmTurquoise2ox was amplified into two inserts to add a synthetic intron of 51 bp (gtaagtttaaacagttcggtactaactaaccatacatatttaaattttcag) between position 211 and 212 along the CDS of the fluorophore. The 5’ CDS insert was amplified with oligos tttcaggtgcccgaaatgtgagctATGGTCTCCAAGGGAGAGGA and tatgtatggttagttagtaccgaactgtttaaacttacCTTGGACTCCCCAGGAGAGGG, while the 3’ CDS insert was amplified with oligos ttcggtactaactaaccatacatatttaaattttcagtcttcgcccgttacccaga and ccgatccccctggcagctagtcttcttgtagagctcgtccattccg. Both SFmTurquoise2ox inserts were generated from the plasmid template pMA-RQ with sequence 21AAF4VD (lot. No: 3026914, invitrogen). The fourth insert (TEV-S::his-24::his-24-3’UTR) was amplified with oligos AAGACTAGCTGCCAGGGG and GGAACAAAAGCTGGGTACCGGGCCCgaagacgttgaacgtcaaattatcaa from the gDNA of the strain MD4331 (*rdvIs1; bcIs148*). The four inserts were inserted into the vector backbone through Gibson Assembly. **pBC1974**: The plasmid pBC1858 was digested with ApaI and EcoRI to generate the vector backbone. The *egl-17* promoter insert was generated from pBC1858 template with oligos TGGATCCCCCGGGCTGCAGGAATTCGATcagatggatgtttactgccaactg and agctcacatttcgggcac. The SFmTurquoise2ox and 3’UTR were amplified from pBC1985 (*Pegl‐17::myristoylatedSFmTurquoise2ox(no intron)::pie-1-3’UTR*) into two inserts to add a synthetic intron of 51 bp (gtaagtttaaacagttcggtactaactaaccatacatatttaaattttcag) between position 174 and 175 along the CDS of the fluorophore. The 5’ CDS insert was amplified with oligos tttcaggtgcccgaaatgtgagc and taatcagggttagttagtatatatatgtttaaacttacccatgggactgggagctttcc. The 3’ insert linked to *pie-1* 3’UTR was amplified with oligos tatatatactaactaaccctgattatttaaattttcagccaaccctcgtcaccacc and ggaacaaaagctgggtaccggg. The three inserts were inserted into the vector backbone through Gibson Assembly. **pBC1999**: The plasmid pBC1590 was digested with NheI and ApaI to generate the vector backbone. The *tomm-20* (N-terminal 54 residues) insert, which contains the linker attB2 (aacccagctttcttgtacaaagtggTCGCCACC), was amplified from pBC1998 (*Ptoe-2::tomm-20(N-term)::mCherry::tbb-2-3’UTR*) template with oligos tcatgtcctcaggtcaaaaGC and GGTGGCGAccactttgtacaag. The mKate2::tbb-2 3’UTR insert was amplified from pBC1858 with oligos tttcttgtacaaagtggTCGCCACCATGTCCGAGCTCATCAAGGAGA and ccgaaacgcgcgagacgaaagggccCTCACCTAGGTATCTAGAACCGG. The two inserts were inserted into the vector backbone through Gibson Assembly.

### Synchronised L1 larval preparation for QL.p imaging

Six L4s are transferred on seeded NGM medium plates (4x) and left to grow at 20 °C for 6 days. By the 6th day, the bacterial lawn is depleted, and many eggs are present. Larvae and adults are washed off using 1 ml MPEG (M9 buffer added with PEG 8000 to a final mass fraction of 0.1% wt (i.e., 1 g in 1 l)) by vigorously shaking plates. After 2–3 washes, residual worms and large particles (>L1 size) present on plates are picked off. Next, plates are incubated at 25 °C for 90 min (first incubation). A small bacterial lawn is prepared on four fresh unseeded NGM plates by resuspending some OP50 from a seeded plate in 80 µl M9 (Na2HPO4 5.8 g, KH2PO4 3 g, NaCl 0.5 g, NH4Cl 1 g, H_2_O to 1 l, sterilised by autoclaving) and applying 20 µl to each plate (hereafter “spread plates”). These plates are shaken to spread bacteria on a larger surface and to make the lawn very thin and any large particle is picked off. After the first incubation, the incubation plates are checked again, and residual L3-adult worms are removed by picking. Next, the newly hatched L1 larvae from the first incubation are washed off from all plates with 2 ml MPEG (the MPEG is transferred plate-by-plate and finally into one 1.5 microcentrifuge tube). L1 larvae are centrifuged at 400 × *g* for 2 min and the MPEG is carefully aspirated off leaving 20 µl containing the L1 larvae. Worms are transferred to a spread plate using a P200 yellow tip. Once the MPEG is completely evaporated, any residual L3-adult worm and particle is removed by picking. The plate are then incubated at 25 °C for 3.5 h (second incubation). At the end of the second incubation, L1 larvae are collected by washing the spread plates using 1 ml MPEG and transferred into a 1.5 microcentrifuge tube and centrifuged at 400 × *g* for 2 min. The MPEG is then carefully aspirated off down to 10–15 µl containing the L1 larvae, which are now ready for mounting.

### Slide preparation

A 10% agarose solution is prepared as follows. In all, 30 ml of 66% M9 (20 ml M9 + 10 ml H_2_O) are added in a 500-ml borosilicate glass bottle and the meniscus level is marked with a permanent marker. Next, 3 g of agarose and additional 40 ml of H_2_O are added before microwaving at low power for as long as needed to reach the marked level (30 ml). To attain the right thickness of the 10% agarose pad on a clean slide (VWR Avantor Slides, Catalogue no. 631-1550), the slide used for mounting is flanked by other two slides taped with two layers of Fisherbrand self-sticking Labelling tape (cat. no. 1590110 G). A drop of the 10% agarose is placed in the centre of the clean slide and covered with another clean slide placed in a perpendicular fashion. Once the pad is cooled (at least 2 min), gently pull aside the slide on top without deforming the pad. The pad is cut into an isosceles triangle (10 mm × 10 mm × 5 mm) and 0.5 µl MPEG containing synchronised L1 larvae are pipetted on the centre. Excess MPEG is evaporated from the pad to avoid excessive dilution of the microsphere suspension later. Meanwhile trying to keep the pad itself from drying out. This is done by placing the slide on top of a refrigerated (4 °C) flat plastic surface (e.g. microcentrifuge rack), to keep the pad cool. Evaporation is done in a 15 °C incubator, where it takes about 2:30 min, but this time changes based on humidity). Care must be taken that the L1 larvae left with enough MPEG to remain hydrated, as well prevent coldshock to the larvae from the refrigerated plastic surface. Next, 0.9 µl of Polybead® microsphere suspension (0.1 μm diameter, 2.5% w/v, Catalogue no. 00876, Polysciences, Inc.) are pipetted on the pad and similarly left to dry out for 1 min at 15 °C (time varies depending on humidity) with the slide in full contact with the refrigerated plastic surface. An 18 mm × 18 mm 1_1/2_ (170 ± 0.005 µm thick) Zeiss coverglass is carefully levered down unto the pad, avoiding air bubbles in the mount. The slide is tilted very gently on both longer sides a few times to compact the coverglass-microsphere suspension-pad system. Ideally, worms are well immobilised on the entire surface of the pad. To prevent the system and larvae from drying out, empty space around the pad is symmetrically filled from opposing sides with mineral oil (Catalogue no. PC5530, Bio Basic), kept at 15 °C, using a cut P200 pipette tip. The mineral oil will create further tension in the system, immobilising the larvae further. Finally, the coverglass is sealed to the slide by gently applying liquid Vaseline to all corners. Worms are now well immobilised everywhere and ready for imaging. All steps requiring manual work should be completed in a short time to prevent the dehydration and consequently the deformation of the agarose pad.

### SR live imaging (*bcIs153*)

We produced SR time-series images on an inverted Zeiss LSM980 with Airyscan2 with a GaAsP-PMT photocathode detector. We used the oil-immersion objective Zeiss C Plan-Apochromat 63×/1.4 Oil DIC M27 (working distance + coverglass: 140 μm + 170 μm = 310 μm). The working Distance information is given in https://www.micro-shop.zeiss.com/en/us/shop/objectives/421782-9900-000/Objective-C-Plan-Apochromat-63x-1.4-Oil-DIC-M27. Worms in the right developmental stage (about 5 h post-hatching at 25 °C) are found using the ZenBlue Locate mode (RFP filter cube) to visualise Q neuroblasts marked with the mCherry markers. Q cells right underneath the coverglass are distinguishable from their counterpart facing the opposite side by their distinctive high contrast and little blur. Once QL.p cells are found to have entered prophase or metaphase, ZenBlue is switched to Acquisition mode and recordings are started. We record every 1 min in “Airyscan SR” mode with a Scan Zoom set to 7.5× (17.96 μm × 17.96 μm or 422 × 422 pixels), 0.0425 μm pixel size, 0.130 μm z-step, and 1.03 μs dwelling time (429.43 ms per frame, in dual colour). The mCherry and mtGFP fluorophores are excited with the 594 nm (0.3% power) and 488 nm (0.4% power) laser lines, respectively. The LSM Scan speed is set to 10, the Scan direction to “bidirectional”, the Detector Gain to 850 V and the Detector Digital Gain to 1.0. In case of QL.p movements that could lead the dividing cell out of the field-of-view, the Image Centre Position is changed between acquisitions to keep the cell centred. When this is not possible, the recording is stopped and QL.p is immediately recentred to start a new recording, on the same division. At the end of each imaging session, czi files are Airyscan-processed (2D mode, standard strength).

### SR live imaging (*bcIs164*)

Worms in the right developmental stage (~5 h post-hatching at 25 °C) are found using the ZenBlue Locate mode (GFP filter cube) to visualise Q neuroblasts marked with the SFmTurquoise2ox markers. Q cells right underneath the coverglass are distinguishable from their counterpart facing the opposite side by their distinctive high contrast and little blur. Once QL.p cells are found to have entered prophase or metaphase, ZenBlue is switched to Acquisition mode, and recordings are started. The imaging system and objective are the same one used to image *bcIs153*-expressing animals. We record every 45 s in “Airyscan SR” mode with a Scan Zoom set to 10× (12.62 μm × 12.62 μm or 358 × 358 pixels), 0.035 μm pixel size, 0.130 μm z-step, and 0.66 μs dwelling time (449.40 ms per frame, in three colours). The SFmTurquoise2ox, GFP and mKate2 fluorophores are excited with the 405 nm (0.5% power), 488 nm (0.7% power), and 594 nm (0.4% power) laser lines, respectively. The LSM Scan speed is set to 14, the Scan direction to “bidirectional, the Detector Gain to 850 V and the Detector Digital Gain to 1.0. In case of QL.p movements that could lead the dividing cell out of the field-of-view, the Image Centre Position is changed between acquisitions to keep the cell centred. When this is not possible, the recording is stopped and QL.p is immediately recentred to start a new recording, on the same division. At the end of each imaging session, czi files are Airyscan-processed (2D mode, standard strength). To the ends of this study, we did not include the GFP tbb-2 channel in the analysis and images illustrated in Supplementary Fig. S8.

### Image processing

For each recording, the following procedure is applied to the last metaphase and post-cytokinesis z-stack images. 2D Airyscan-processed. czi files are opened in Fiji (ImageJ 1.53f51)and the two channels are split. The red channel z-stack (QL.p plasma membrane and chromatin) is aligned by registration with MultiStackReg plugin^69^ and the alignment output is saved as a TranformationMatrices.txt file. The.txt file is loaded on the same plugin window for the alignment of the green channel z-stack (QL.p mitochondria). Channels are then merged and a smaller z-stack including mostly QL.p is cropped and saved. Next, to account for bleedthrough, channels are split again, and the red channel is subtracted from the green one. We also performed an alternative subtraction by prior multiplication of the mCherry channel by 0.12 as described in the main text. No subtraction was performed of images generated from animals expressing *bcIs164* as the SFmTurquoise fluorescence signal does not bleed through the mKate2 channel. After subtraction, channels were deconvolved using DeconvolutionLab2^70^. Both red and green z-stacks are deconvolved using two theoretical and symmetric (no spherical aberration) PSFs that model the same 3D light diffraction pattern but differing in the emission peak wavelengths (474 nm for SFmTurquoise2ox, 510 nm for mtGFP, 610 nm for mCherry, and 633 nm for mKate2). The PSFs were computed on the Diffraction PSF 3D plugin (https://www.optinav.info/Diffraction-PSF-3D.htm). The Rayleigh resolutions are predicted to be 4.78 pixels (SFmTurquoise2ox), 5.14 (mtGFP), 6.15 pixels (mCherry), and 5.38 pixels (mKate2). The deconvolution is performed applying the Richardson–Lucy (RL) algorithm^71,72^ with 20 iterations. Channels are then merged, and voxel sizes are redefined as originally set in the “image properties” window. Images are now ready for 3D rendering.

### 3D rendering

Deconvolved z-stacks are opened in Imaris 9.8 and both channels are rendered using the Surface rendering function. Cell shapes in the red channel are manual rendered drawing ROIs on each slice, while mitochondria are automatically rendered. For manual rendering of cell surfaces, we used a Wacom graphic board equipped with a wireless digital graphic pen. For surfaces of mitochondria, we used the following setup with automatically defined thresholds:

#### Algorithm

- Enable Region of Interest = true
- Process Entire Image = false
- Enable Region Growing = false
- Enable Tracking = false
- Enable Shortest Distance = true

#### Region of interest

- Region1: XYZT from [X Y Z T] to [X Y Z T]

#### Source channel

- Source Channel index = 2 (green channel)
- Enable Smooth = true
- Surface Grain Size = 0.00100 μm
- Enable Eliminate Background = true
- Diameter Of Largest Sphere = 0.319 μm (mtGFP) or 0.264 μm (tomm-20(N-term)-mKate2)

#### Threshold

- Enable automatic threshold = true
- Manual Threshold Value = 2544.68 (example)
- Active Threshold = true
- Enable Automatic Threshold B = true
- Manual Threshold Value B = 22176.6 (example)
- Active Threshold B = false

Both cell and mitochondrial shapes are then split into either anterior/posterior surfaces or QL.pa/QL.pp surfaces at metaphase and post-cytokinesis, respectively. Data for statistical analysis are then exported into excel files.

## Methods (protocol validation)

### Determination of the apparent distance between the coverglass and QL.p

We assumed the apparent imaging plane to be the centre of the range spanning the top-to-bottom dimension of QL.p. The acquisition mode is set to LSM (confocal mode), and the reflection mode is active in ZenBlue. The scan zoom is set to 7.5× (211 × 211 pixels or 17.96 μm × 17.96 μm), both laser lines set to 0.2% power, laser blanking is enabled, the dwelling time is 2.06 μs and the frame time is 107.36 ms. The LSM scan speed is set to 10, the scan direction is bidirectional and both line step and averaging are 1. The scaling per pixel is 0.085 μm × 0.085 μm, the detector gain is 989 V, the detector digital gain is 1.0, the detector offset is 0. With this setup, the acquisition live mode is started, and the control knob of the nosepiece is scrolled up and down in z to find the centre of QL.p. Once the centre is found, the z position is recorded, and the scanned field is moved laterally far away from the cell without changes in *z*. Next, the nosepiece is moved again to find the water-coverglass interface. This can be recognised by the typical increase in average pixel intensity (reflection of the excitation light back to the detector), which is visibly higher than the intrinsic detector noise noticed by focusing on any other depth. This procedure was repeated three times on three difference QL.p cells found in ideal positions for time-series imaging. The distance between each of them and the coverglass was always around 2 μm. That value was chosen as the CG-APP distance (Supplementary Fig. S5c) (distance between the coverglass and the apparent image plane of QL.p) to calculate the longitudinal spherical aberration.

### Calculation of the physical distance between coverglass and QL.p

The Θ angle (blue in Supplementary Fig. S5c, e), which is used to define the NA of any microscope objective, measures 32.63° and is calculated as the arctangent of the ratio between the opposite r max side (radius of the objective lens, 198.5 μm) and the adjacent OL-APP side (working distance of the objective + coverglass: 310 μm) (Supplementary Fig. S5e, i). The accuracy of this calculation is verified by reproducing the objective NA value (1.40), which is the sine of 32.63°*2 (two Θ angles include all the light captured by the objective lens) multiplied by the Zeiss Immersoil^TM^ RI (1.518). By using the Snell’s law^73^, the refractive angle (Θr, red) is about 36.81°, and is calculated as the arcsine of the product between the sine of Θ (0.54) and the 1.1095 (i.e., the ratio between the RI of coverglass (1.52) and that of *C.elegans* (here assumed to be 1.37)^74–76^) (Supplementary Fig.S5e, i). Next, the Θ’ angle (orange) measures 57.37° (90°-Θ angle) and is used to calculate H (2.381 μm), which is the ratio between GC-APP (2 μm) and sine of Θ’ angle (0.84)). As a result, H is multiplied by the sine of Θ (0.54) to obtain L, which is 1.286 μm (Supplementary Fig. S5e, ii). Next, H’ is 2.137 μm and is the ratio between L and the sine of Θr (0.60) (Supplementary Fig. S5e, iii). The Θ” angle (green) measures 53.19° (90°-Θr angle) and its sine (0.80) is multiplied by H’ to calculate CG-ACT (1.710 μm), which is the true distance between the centre of QL.p and the coverglass (Supplementary Fig. S5e, iv). Therefore, the average positive longitudinal spherical aberration is 0.290 μm (CG-APP-CG-ACT). Next, the same mathematical procedure was used to calculate the positive longitudinal spherical aberration at the top and bottom focal planes of QL.p, which are assumed to be 0.5 μm and 3.5 μm far from the coverglass, respectively. These distances were assumed considering QL.p to be 3-μm thick and its centre to be 2 μm far from the coverglass (apparent image plane APP) (Figs. S5c, f and S2). The positive longitudinal shift (LS) increases proportionally to the increase in the distance between the coverglass and the focal plane (Supplementary Fig. S5f, right). These values were used to estimate the volume of QL.p (Supplementary Fig. S5f, right), which is simplified as an ellipsoid, using the apparent volume of QL.p (Supplementary Fig. S5f, left).

### Determination of mCherry and mtGFP brightness

We opened Airyscan-processed images in Fiji and split channels. Both mCherry and mtGFP brightness (mean Integrated Density) were calculated by selecting only the central section crossing QL.p daughters at post-cytokinesis. Next, measurements (on the main toolbar: analyse->set measurements) included Area and Mean grey values. We checked off the “Limit to Threshold” box in the same “set measurements” window. Then, we performed autothresholding for both mtGFP and mCherry images (Default algorithm), and we measured the parameters within the segmented areas.

### Analysis of photobleaching and mitochondria quantity change

To determine photobleaching rates, all time points of time-series images were analysed. For each mtGFP z-stack, from which the mCherry channel had been subtracted to remove bleedthrough, ROIs were drawn in Fiji along QL.p plasma membrane on all focal planes intersecting QL.p. The total mtGFP fluorescence was measured as the sum of all Integrated Densities (IntDen) measured on each ROI along metaphase and post-cytokinesis z-stack. For the multi-step time-series analysis, changes in QL.p mitochondrial quantity during cell divisions were determined by measuring both the mtGFP IntDen (in fully processed images, Fiji) and the mitochondrial volume (in 3D rendered images, Imaris) from the same metaphase and post-cytokinesis z-stacks. Regarding the two-step time-series analysis, the same measurements of mtGFP IntDen were conducted on fully processed images that were taken only during QL.p metaphase and post-cytokinesis.

### Generation of image histograms

To generate image histograms, we used metaphase and post-cytokinesis images either processed in Fiji or unprocessed (original Airyscan-processed.czi images). For the former, z-stacks matching the volumes of interest (VOIs), which were manually determined in Imaris during 3D rendering of mitochondria, were used. In both cases, we split z-stacks in Fiji and only images of mitochondria were considered. On the main toolbar in Fiji, we selected “analyse” and then “histogram” (alt+H). For relative histograms, we used the following setup: bins=256, X_min_=0, X_max_= given value, Y_max_=auto, check off “stack histogram”. For fixed histograms, we used the following setup: bins=256, X_min_=0, X_max_= 30000 (processed images in Fiji) or 7300 (original Airyscan images), Y_max_=auto, check off “stack histogram. After selecting OK, we extracted data from histogram lists and copied them into excel files. We summed up the 256 counts and divided each count by their sum to generate count frequencies for all the 256 bins.

### Analysis of threshold algorithms

To analyse threshold algorithms in Fiji, we used metaphase and post-cytokinesis images processed in Fiji, which were split and only images of mitochondria were considered. We used the threshold window (Image->adjust->threshold) in Fiji and selected the desired threshold algorithm. We checked off “stack histogram” and pressed “auto”. Next, we opened the histogram window (alt+H) and setup the analysis on processed z-stacks matching the VOIs in Imaris as it follows: untick “use pixel value range”, check off “stack histogram”, bins=30, X_min_=automatic threshold value given the threshold window, X_max_= given value, Ymax=auto. After selecting OK, we extracted data from histogram lists and copied them into excel files. We summed up the counts (=pixel content of mitochondrial objects) in the 30 bins.

### 2D-rendering of mitochondria using the Isodata algorithm

To analyse mitochondrial quantities with the IsoData algorithm in Fiji, we used metaphase and post-cytokinesis images processed in Fiji. Z-stacks matching the volumes of interest (VOIs), which were manually determined in Imaris during 3D rendering of mitochondria, were split in Fiji. After ticking the “freehand selections” on the main toolbar, we drew ROIs along contours of the cell (mCherry channel) on all relevant focal planes and saved them as.zip file. We then selected the respective mitochondrial image and opened the threshold window (Image->adjust->threshold). We checked off “stack histogram”, “dark background” and “don’t reset range”. We then selected “IsoData” in the dropdown menu and “apply”. We clicked “convert to (8-bit) mask” in the threshold window. In the “convert stack to binary” window, we checked off “ calculate threshold for each image”. The threshold window is not closed during the next steps. On the main toolbar, we selected “Edit->invert(all images)” and saved the new binary image as.tif image. Next, we open the.zip file containing the ROIs of the cell, and, on the main toolbar, we selected “plugin->macros->run->find the following macro (.txt file extension):

n=roiManager(“count”);

for(i = 0; i < n; i + +){

roiManager(“Select”, i);

run(“Make Inverse”);

setBackgroundColor(0, 0, 0);

run(“Clear”, “slice”);

}

Next, after closing ROImanager, we duplicated the new stack (CTRL + SHIFT + D) by typing in “range” the bottom and top slices on which we previously drew the last and first ROIs appearing in the ROImanager list. Then, the image is saved as.tif file. On the threshold window, we clicked “auto” and then, on the main toolbar, we checked off “area” in “analyse->set measurements”. Next, we processed all images through “analyse->analyse particles (check off “pixel units”)->OK”. We saved the ROIs generated in ROImanager and copied the results on an excel datasheet. All areas were summed up (=mitochondrial quantity in 2D) and multiplied the result by the pixel content of a μm^2^ (553.63 pixels).

### Structured-illumination microscopy and 3D rendering of QL.p mitochondria

We used a Zeiss Elyra 7, upgraded with the SIM^2^ image reconstruction algorithm and with dual sCMOS cameras for simultaneous acquisition of two channels, to image QL.p mitochondria in timeseries. We used the oil-immersion objective Zeiss alpha Plan-Apochromat 63×/1.46 Oil Korr M27 (working distance + coverglass: ~130 μm + 170 μm = ~300 μm). The working Distance information is given in https://www.micro-shop.zeiss.com/en/us/shop/objectives/420780-9971-000/Objective-alpha-Plan-Apochromat-63x-1.46-Oil-Corr-M27. Worms in the right developmental stage (about 5 h post-hatching at 25 °C) are found using the ZenBlue Locate mode (RFP filter cube) to visualise Q neuroblasts marked with the mCherry markers. Q cells right underneath the coverglass are distinguishable from their counterpart facing the opposite side by their distinctive high contrast and little blur. Once QL.p cells are found to have entered prophase or metaphase, ZenBlue is switched to Acquisition mode and recordings are started. In Supplementary Fig. S14a, S14b, we recorded every 1 min in lattice SIM (9 phases for each of the 37 sections) with 3D leap mode with a FOV of 11 μm × 10.01 μm (or 350 × 322 pixels), 0.031 μm pixel size, 0.110 μm z-step, and 50 ms exposure time for both colours (camera temperature set to 7 °C). The acquisition of individual z-stacks takes about 20 s. The mCherry and mtGFP fluorophores are excited with the 561 nm (0.4% power) and 488 nm (0.4% power) laser lines, respectively. After image reconstruction, the voxel size is 0.031 × 0.031 × 0.037 µm (from 37 to 111 sections along the optical axes).

In Supplementary Fig. S14c, S14d, we generated single time point images in apotome SIM (5 phases) with 3D leap mode with a FOV of 80.14 μm × 80.14 μm (or 1280 × 1280 pixels), 0.063 μm pixel size, 0.329 μm z-step, and 80 ms exposure time for both colours (camera temperature set to 7 °C). The acquisition of large FOVs (4.93 µm deep that means 16 sections) takes about 8 sec. The mCherry and mtGFP fluorophores are excited with the 561 nm (0.7% power) and 488 nm (1.1% power) laser lines, respectively. We then applied SIM^2^ image reconstruction as it follows. We set the advanced Filters with Fast Fit, Gauss, without detrending, 15 iterations, and 0.08 regularisation weight. After image reconstruction, the voxel size is 0.032 × 0.032 × 0.110 µm. Images (.czi format) were directed converted into.ims files and 3D rendered in Imaris as described above apart from the Surface Grain Size (=0.0626 µm) and the Diameter Of Largest Sphere (=0.235 µm).

### Statistical analysis

All statistical analyses provided for results illustrated in Figs. 1–4 and in Supplementary Figs. S1–S13 were conducted in GraphPad Prism 8.3.0, except for results in Supplementary Fig. S10a, S10b, which were conducted in R (see below). For linear regressions (*y* = b*x + *a*, where *a*=intercept and *b*=slope, *x*=metaphase data, *y*=post-cytokinesis data), average grey value frequency profiles were log_10_-log_10_ transformed and then a linear regression was fitted according to the least square regression method (1000 iterations maximum). We defined initial values for both parameters: *a* = 0, *b* = 1. We did not weight values and minimised the sum-of-square of the distances of the points from the regressions. To compare the slope from the hypothetical *b* = 1 value, we performed an extra-sum-of-squares *F*-test without constraining the parameter. The analysis of grey value frequency distributions for individual divisions was carried out in R 4.2.2 and is detailed in full in the supplementary method.

### Illustrations

Figures and schematics were generated in Affinity Designer (versions 1.7.3 and 2.4.1) and R 4.2.2.

## Supplementary information

Supplementary information

Supplementary method

Supplementary Movie 1

Supplementary Movie 2

## Data Availability

The data sets generated as part of this study are available from the corresponding authors upon request.
