# Author Correction: Impact of photobleaching on quantitative, spatio-temporal, super-resolution imaging of mitochondria in live *C. elegans* larvae

**DOI:** 10.1038/s44303-025-00102-1

**Published:** 2025-08-02

**Authors:** Ioannis Segos, Jens Van Eeckhoven, Alan Greig, Michael Redd, Christopher Thrasivoulou, Barbara Conradt

**Affiliations:** Research Department of Cell and Developmental Biology, Division of Biosciences, The Centre for Cell and Molecular Dynamics, University College London, London, UK

**Keywords:** Biological techniques, Cell biology

Correction to: *npj Imaging* 10.1038/s44303-024-00043-1, published online 06 November 2024

In this article, author names were presented in an incorrect family name, given name order. The original article has been corrected.

