# Supplementary material for: Impact of photobleaching on quantitative, spatio-temporal, super-resolution imaging of mitochondria in live C. elegans larvae
Source: Npj Imaging. 2024 Nov 6;2:43. doi: 10.1038/s44303-024-00043-1 (PMC11541191; doi:10.1038/s44303-024-00043-1)
Supplement: Supplementary file 1 — Supplementary information [file 44303_2024_43_MOESM1_ESM.pdf]

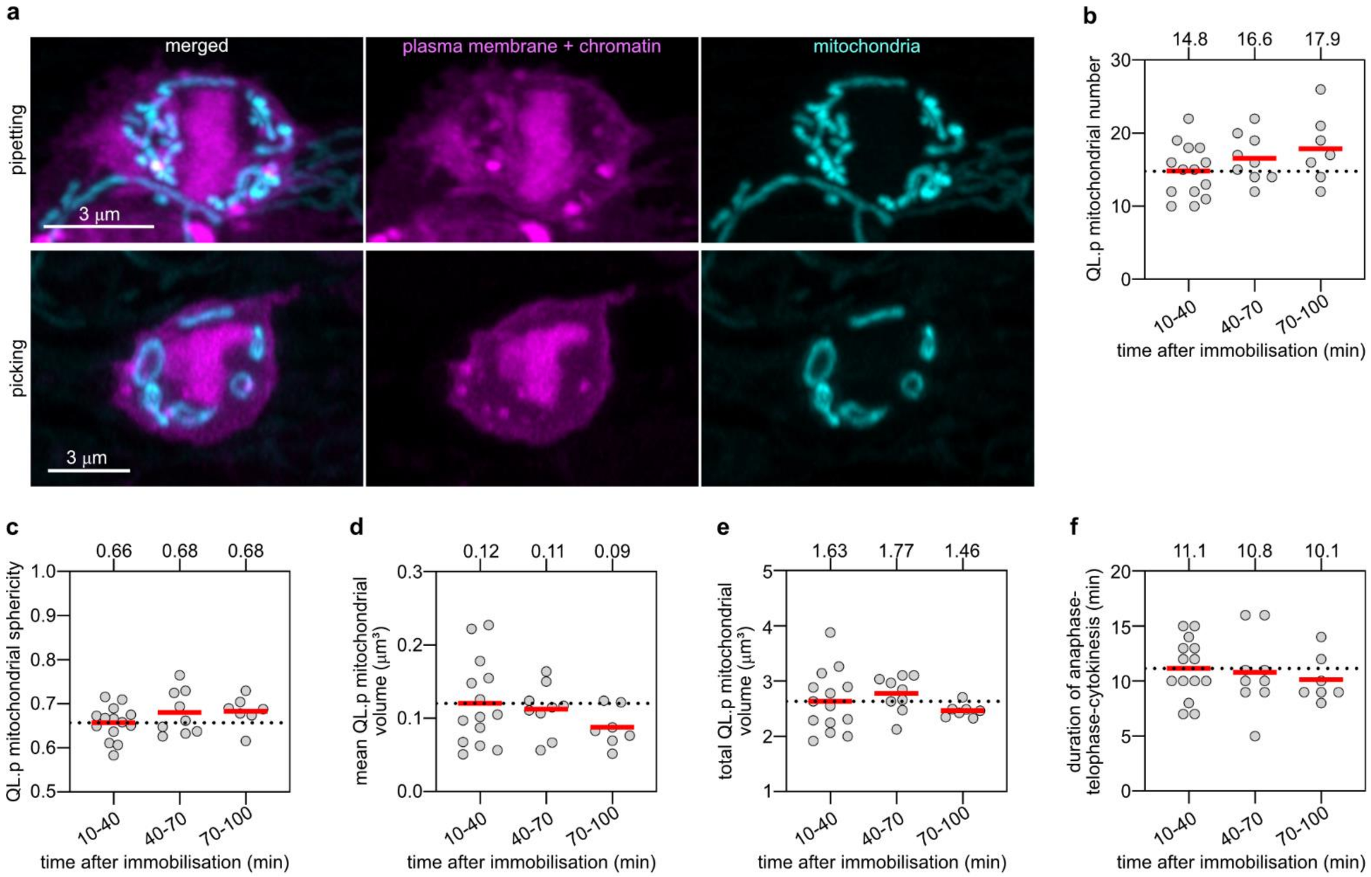

Fig.S1 | Worm pipetting improves the health status of L1s during imaging. a, Signs of acute stress (circular mitochondria) are generally visible only when L1s are picked (bottom row) on the agarose pad (top row: normal mitochondrial morphology and metaphase). In stressed animals expressing *bcIs153*, QL.p mitochondria are often circular (bottom left and right), and chromosomes fail to align along the metaphase plate (bottom left and centre). Left: merged; centre: QL.p plasma membrane and chromatin markers (mCherry); right: mtGFP (cyan). **b**,**c**,**d**,**e**,**f)** Analysis of QL.p mitochondria and cell division parameters over time, under immobilisation conditions. Each data point refers to individual QL.p divisions. Mitochondrial parameters in **b**, **c**, **d** and **e** and were extracted from 3D rendered mitochondrial images (last metaphase time point) in Imaris. Duration of QL.p division in **f** was measured as the numbers of z-stacks being between the last metaphase time point and the end of cell division (post-cytokinesis). Sorting into the three "time after immobilization" classes was based on the time between mounting and the beginning of cell division. P values are calculated using an ordinary one-way ANOVA with Benjamini, Krieger and Yekutieli correction (**b**,**c**,**d**,**e** and **f**). Normality was tested with the Shapiro-Wilk test. Red bars = mean (**b**,**c**,**d,e** and **f**). Dotted lines are fitted to the averages at 10-40 minutes post-immobilisation. n=14 (10-40min); n=9 (40-70min); n=7 (70-100min) in **b**,**c**,**d**,**e** and **f**.

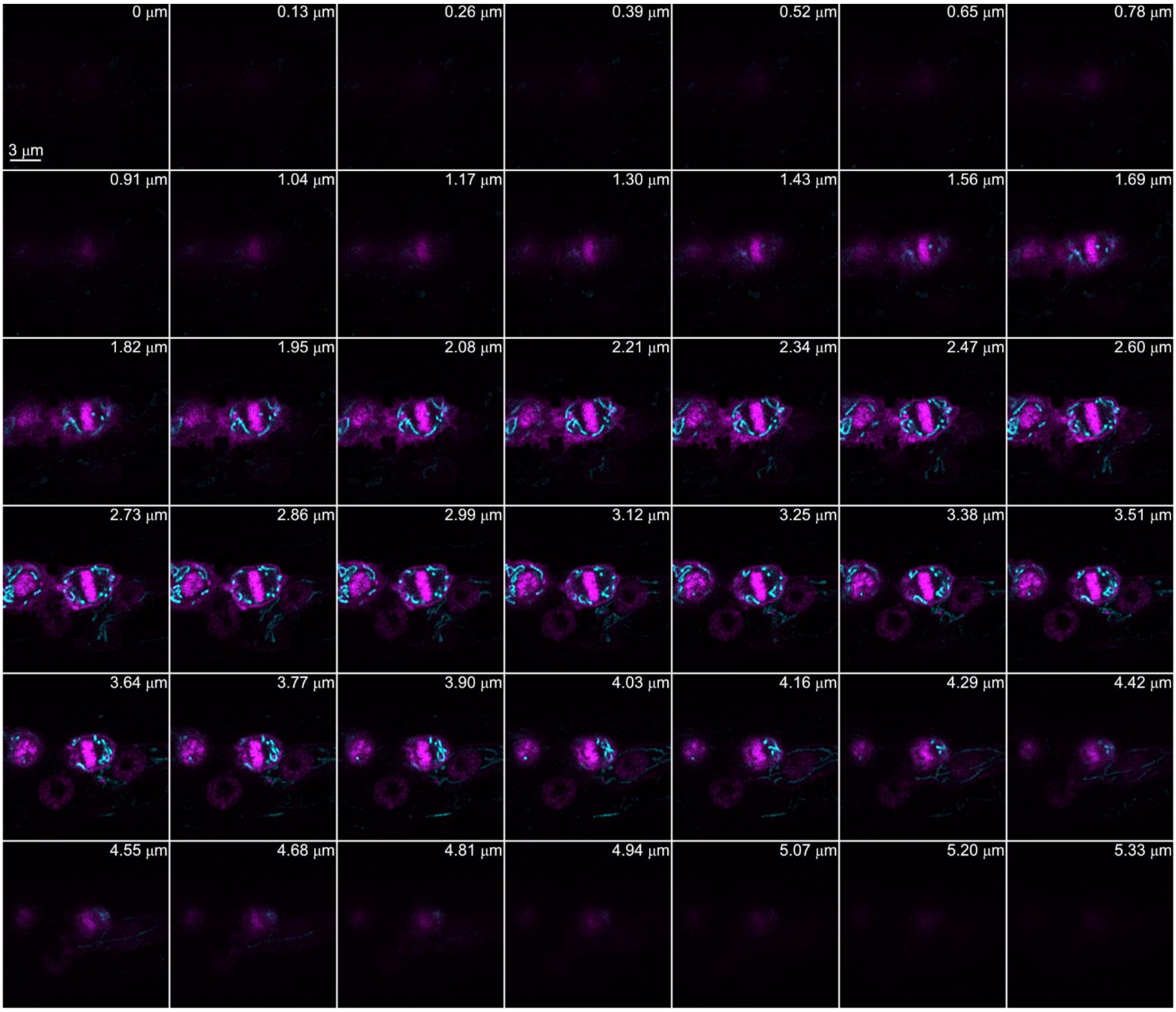

Fig.S2 | Acquisition of z-stacks in time series during QL.p division. Showed here is a 2D Airyscan-processed z-stack (no additional processing in Fiji) generated by imaging QL.p in animals expressing *bcIs153*. QL.p is sectioned at each time point along the optical axes to generate z-stacks. QL.p is oversampled in z (z-step of 130nm instead of 170nm (see methods)) to improve the deconvolution output. The z-stack range can vary between time series and is set with additional sections above and below the cell to accommodate potential movements of the cell along the optical axes. Furthermore, the field of view (FOV) is larger than the cell to include QL.p in every section in case of lateral movements during acquisition. Top right corners on each section give z-positions. This z-stack represents the original image used to illustrate the time course and image processing illustrated in Fig.2 (subfigures a (time -1min), c, d and e) and Fig.3 (subfigures a-f).

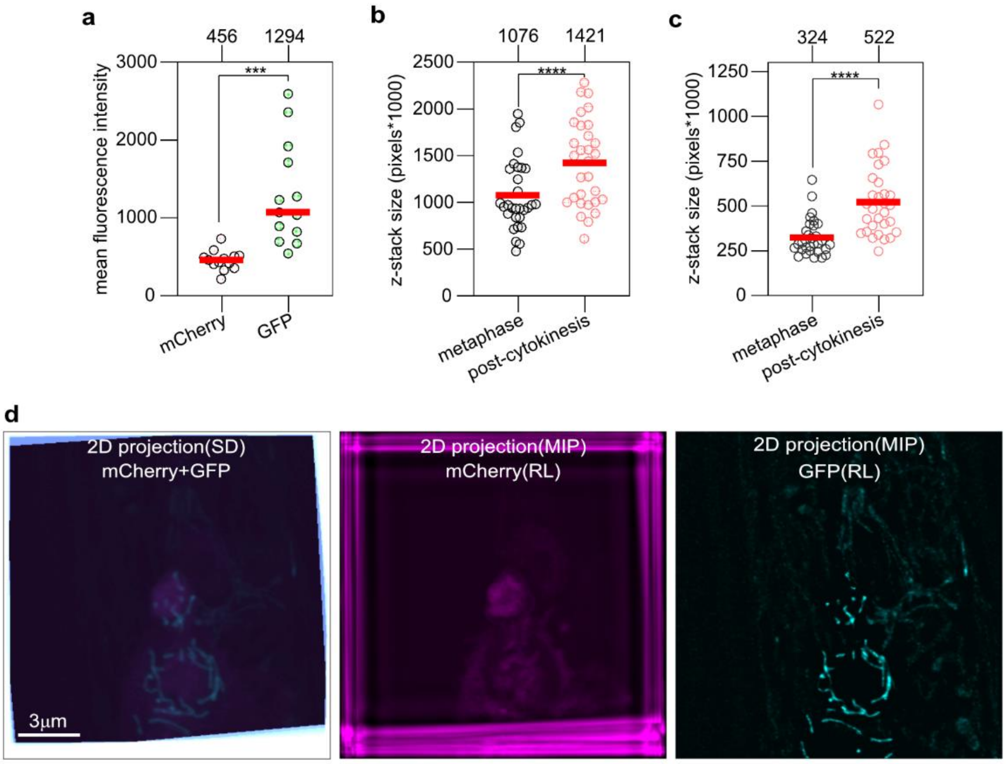

Fig.S3 | Adjustment of image processing: channel subtraction and image cropping. a, Mean fluorescence intensity (Integrated Density) measured on the slice sectioning the central plane of QL.p daughters based on the "Limit to Threshold" option in Fiji (*n* = 13). Automated Default threshold values were applied to both mCherry (myristoylated mCherry + mCherry::his-24) and GFP (mtGFP) images. **b**, The size of the z-stack is the pixel content of volumes cropped during image processing in Fiji before image deconvolution (see also Fig. 2d) (*n*=30). **c**, The size of the z-stack is the pixel (= voxel) content of volumes defined to 3D render mitochondria in Imaris (*n*=30). **d**, Z-stack alignment produces translations and rotations of slices relative to each other (left, s*tandard deviation* (SD) projection) (see also Fig. 2b). Deconvolution of uncropped aligned z-stacks produces artefacts in the mCherry image (centre, bright lines on every side), but not in the GFP image (right). The central and right panels are *maximum intensity* projections (MIP). Images are from a QL.p cell in animals expressing *bcIs153*. The P value is calculated performing a Wilcoxon matched pairs signed rank test (**a,b** and **d**). Normality was tested with the Shapiro-Wilk test. *: P value ≤ 0.05; **: P value ≤ 0.01; ***: P value ≤ 0.001; ****: P value ≤ 0.0001.

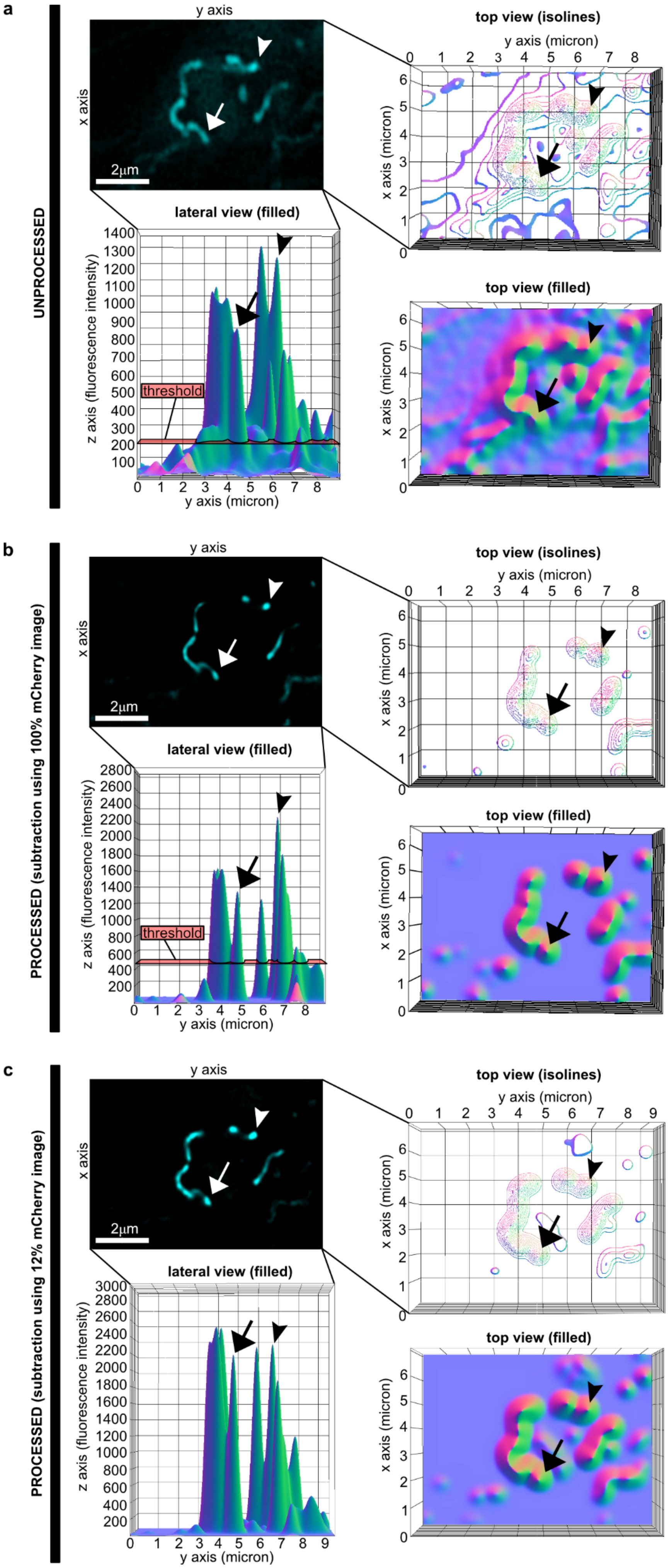
a
UNPROCESSED
y axis
x axis
2μm
top view (isolines)
y axis (micron)
x axis (micron)
lateral view (filled)
z axis (fluorescence intensity)
threshold
y axis (micron)
top view (filled)
x axis (micron)
b
PROCESSED (subtraction using 100% mCherry image)
y axis
x axis
2μm
top view (isolines)
y axis (micron)
x axis (micron)
lateral view (filled)
z axis (fluorescence intensity)
threshold
y axis (micron)
top view (filled)
x axis (micron)
c
PROCESSED (subtraction using 12% mCherry image)
y axis
x axis
2μm
top view (isolines)
y axis (micron)
x axis (micron)
lateral view (filled)
z axis (fluorescence intensity)
y axis (micron)
top view (filled)
x axis (micron)

Fig.S4 | Image processing improves threshold-based segmentation of mitochondria. Illustration of the effects of the image processing methods utilized to enhance the signal-to-background contrast. **a**, In unprocessed images, mitochondrial objects are characterized by moderately sharp grey value peaks with a considerable background and mCherry signal represented together by multiple grey value isolines (top view isolines). **b,c**, In processed images, mitochondrial objects are characterized by very sharp grey value peaks with a “flat” background (purple in the top view (filled)) showing nearly no grey value isolines (top view isolines). Image processing was performed through image subtraction using 100% (**b**) or 12% (**c**) of the mCherry fluorescence intensity (see main text). Lateral and top view images were generated using the 3D Surface Plot function in Process (ImageJ). The 12% fluorescence intensity (panel **c**) was calculated considering both different laser powers and the wavelength to excite mCherry. We excited mCherry by 87% using a 594nm laser, while mCherry is maximally excited at 587nm. The 594nm laser for mCherry was set to 0.3% power, while the 488nm laser power for mtGFP was set to 0.4% (1.33 more power). Therefore, we calculated that 8% of the theoretical 100% (maximal excitation at 587nm) equals to approximately 9% exciting mCherry by 87%. Therefore, we used 9% value and multiplied it by 1.33 to reproduce the theoretical bleedthrough (12%). Arrows and arrowheads point to the same respective grey value peaks shown in lateral and top view images. Fiji IsoData threshold values are reported in the “lateral view (filled)” 3D plots in **a** and **b**.

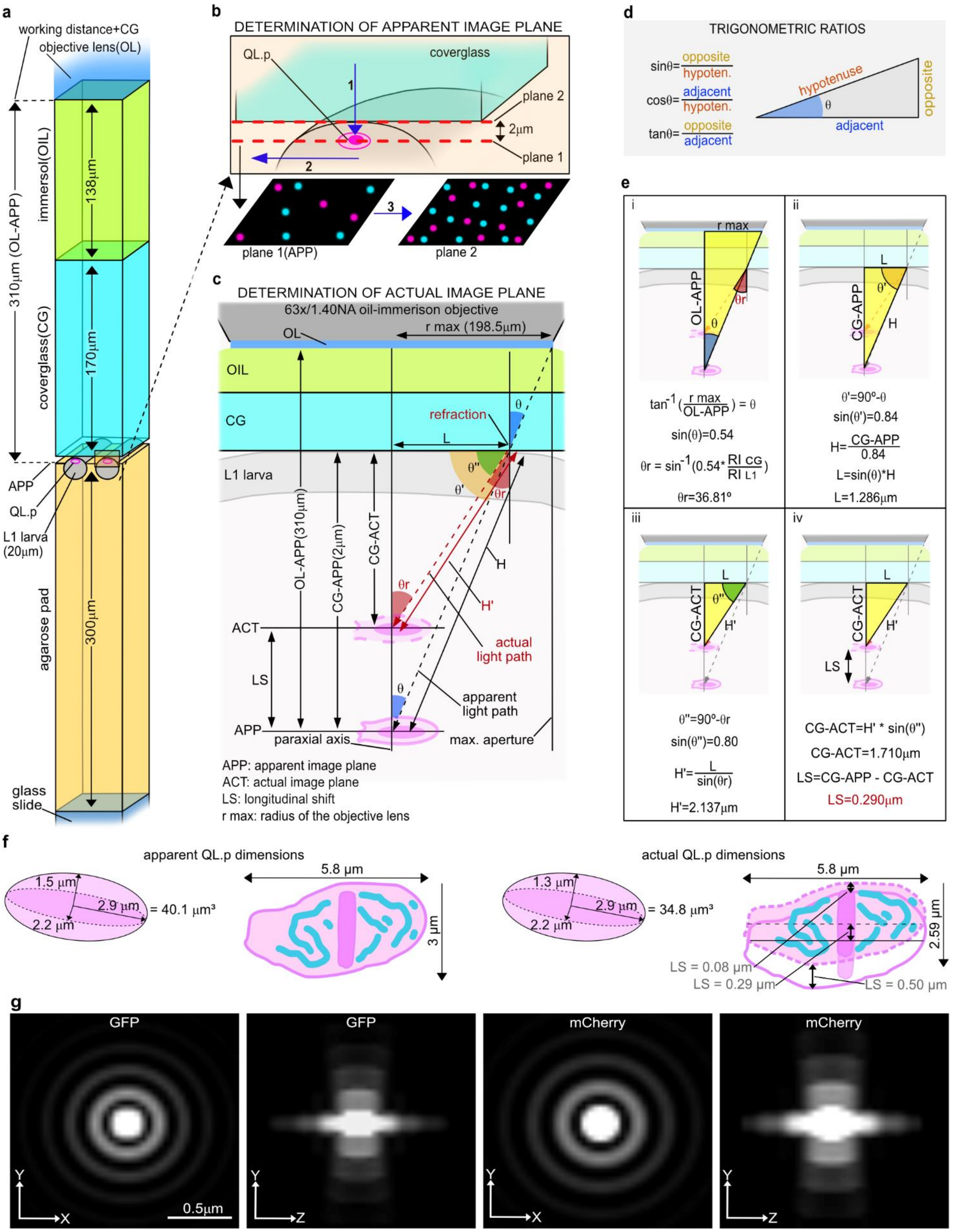

Fig.S5 | Longitudinal spherical aberration and PSF computation. **a**, Cartoon vertical section through glass slide, agarose pad, coverglass and immersion oil with immobilised L1 larvae during imaging. The image shows true sizes of relevant components in the imaging system. Acronyms refer to Fig. S5c. **b**, Determination of the distance between the apparent image plane and the coverglass in three steps: 1) focus on the central plane of QL.p (plane 1), 2) move the field of view away (same z position), and (3) focus on the coverglass-immersion oil interface (plane 2). In active reflection mode, the laser beam is partially reflected and detected focusing on plane 2. **c**, 2D cartoon of the imaging system showing all relevant dimensions and angles to calculate the longitudinal spherical aberration (LS). The components of the imaging system, in this representation, do not have true dimensions, but refer to those given in Fig. S5a. **d**, The three primary trigonometric ratios. **e**, The four steps to measure the average longitudinal

spherical aberration (LS) at QL.p central plane (APP) are based on trigonometric ratios and the sine rule. Cartoons in each panel refer to Fig. S5c. RI_{CG} and RI_{L1}= refractive indexes of the coverglass-oil layer and L1 larvae (1.52 and 1.37, respectively); θr = angle of refraction. **f**, Apparent and actual QL.p indicative dimensions (ellipsoids) assuming top and bottom planes of QL.p apparently at 0.5 μm and 3.5 μm away from the coverglass, respectively (apparent QL.p thickness is about 3 μm, see also Fig.S2). The apparent QL.p cell is thicker (3 μm) than it really is (~2.6 μm). **g**, Determination of GFP (left) and mCherry (right) Point Spread Functions (PSF). For the two PSFs, both top (XY) and lateral (YZ) views are given. No longitudinal spherical aberration (symmetry in YZ) was included.

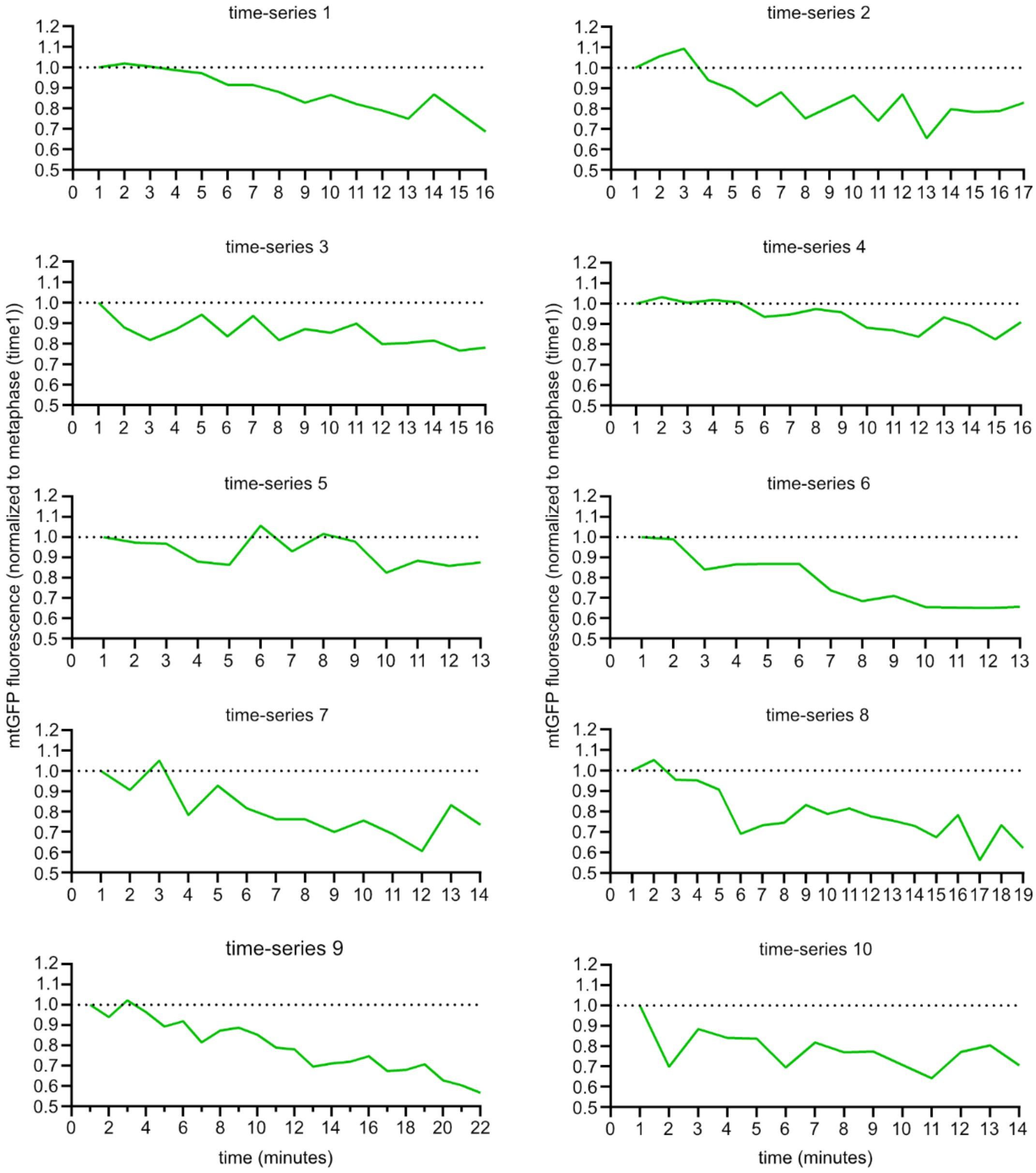

Fig.S6 | Stochasticity of mitochondrial matrix-targeted GFP fluorescence during live imaging. Time series representing ten QL.p divisions highlighting mtGFP fluorescence intensity profiles over time. Each time point is normalized to time 1, while time axes span the entire duration of time series. These individual profiles are showed together in Fig. 4c.

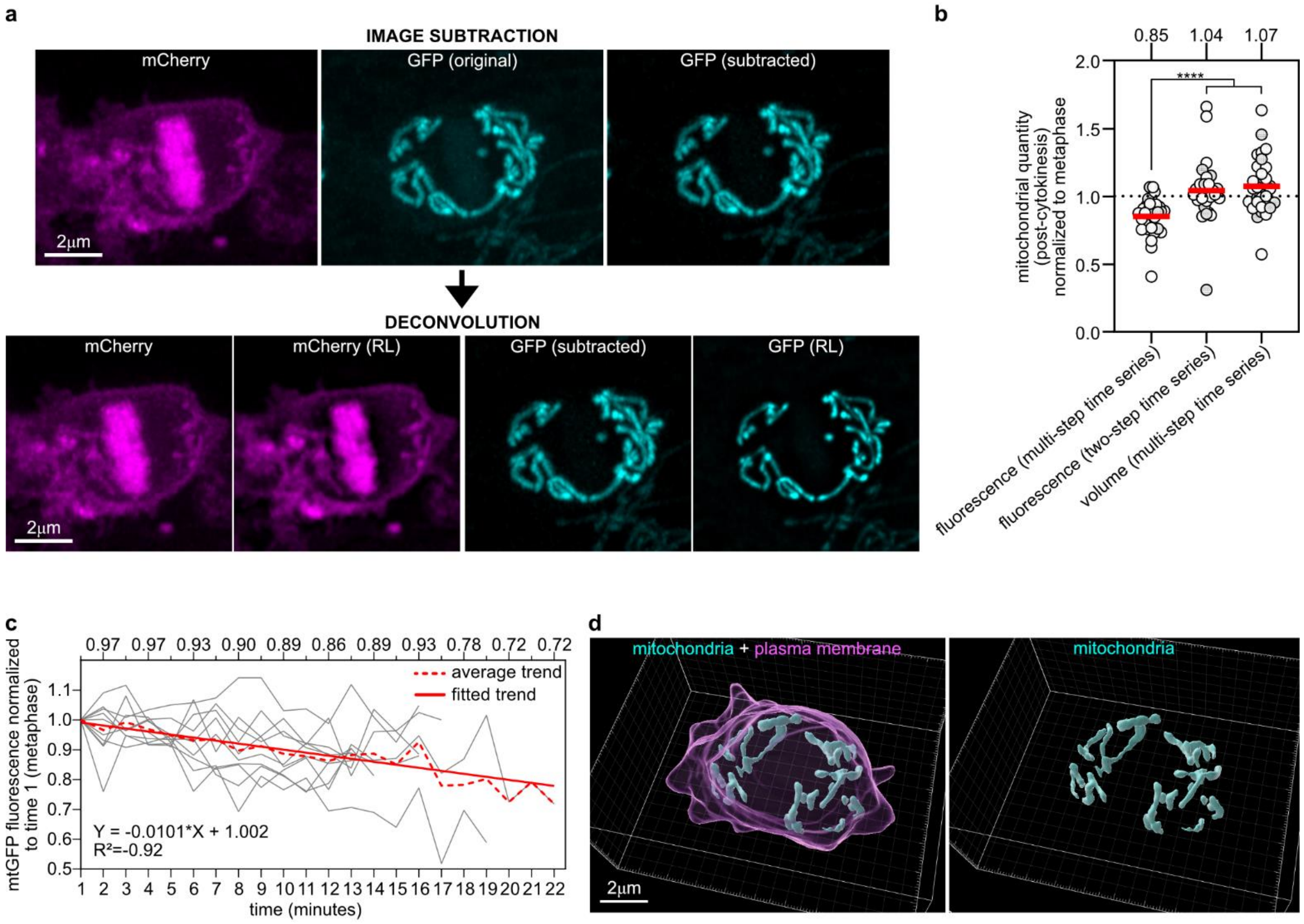

Fig.S7 | Image processing through 12% mCherry subtraction reproduces the ineffectiveness of photobleaching in interfering with threshold-based segmentation of mitochondria. **a**, Effects of image subtraction using 12% of the mCherry fluorescence intensities. Image subtraction (top): 12% of the signal obtained in magenta z-stacks (plasma membrane + chromatin (mCherry), left) are subtracted from cyan z-stacks ('GFP (original)', centre). This arithmetic operation has no effects on mtGFP signal distribution ('GFP (subtracted)'). The subtraction is conducted on the entire z-stack (see also Figure 2c). Next, effects of image subtraction by using 12% mCherry fluorescence intensity during image deconvolution are illustrated on the bottom row (see also Figure 2e). **b**, Mitochondrial quantities (post-cytokinesis) normalized to respective quantities at metaphase (*n* = 30, 24, 30) using processed images through image subtraction using 12% of the mCherry fluorescence intensities. See Figure 4a for more information about data preparation. The fluorescence decay (on average 15%) of mtGFP along multi-step time-series is significantly different from 1 (one sample Wilcoxon test, median = 0.88, P value<0.0001). There is no significant change in quantity (fluorescence) either by taking only two z-stacks (two-step time-series) (average normalized fluorescence intensity: 1.040±0.248sd) (one sample Wilcoxon test, median = 1.01, p-value=0.35) or by segmenting mitochondria in 3D (Imaris) using multi-step timeseries (average normalized volume: 1.073±0.210sd) (one sample Wilcoxon test, median = 1.06, p-value=0.10). **c**, Average mtGFP photobleaching (slope = -0.0101, 95% confidence interval = -0.01312 to -0.007149) from trends constructed on fluorescence values normalized to time 1 using processed images through image subtraction using 12% of the mCherry fluorescence intensities (*n* = 10, referring to Fig. 4a top). Individual photobleaching trends show fluctuations as large as 25% between consecutive time points. **d**, Overview of QL.p cell and mitochondria 3D models in Imaris. Images were rendered using processed images through image subtraction using 12% of the mCherry fluorescence intensities. P values are calculated using a Kruskal-Wallis non-parametric rank test with Benjamini, Krieger and Yekutieli correction (**b**). Normality was tested with the Shapiro-Wilk test. *: P-value ≤ 0.05; **: P-value ≤ 0.01; ***: P-value ≤ 0.001; ****: P-value ≤ 0.0001. Red bars in b = averages.

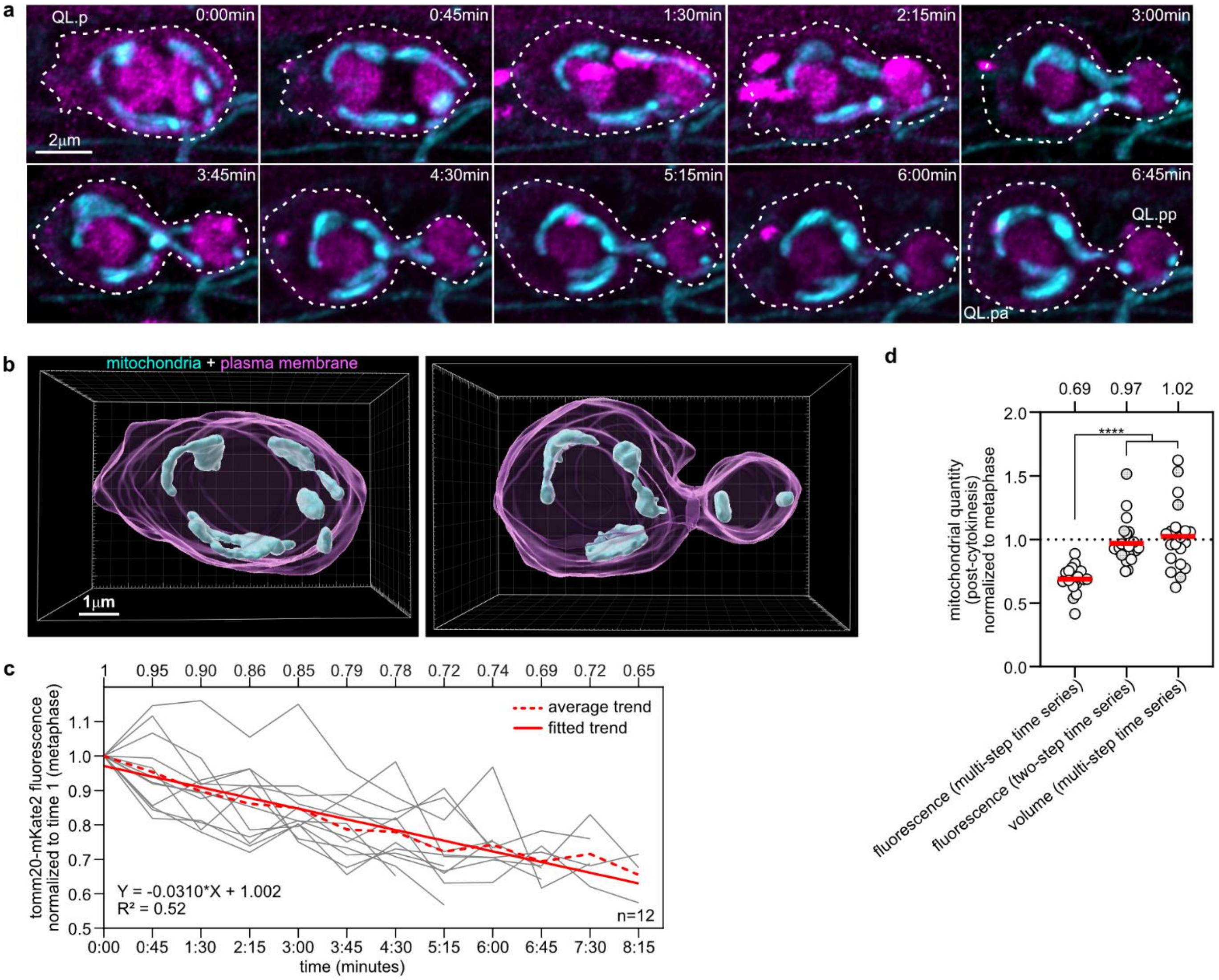

Fig.S8 | Photobleaching does not interfere with threshold-based segmentation of mitochondria in Imaris using TOMM-20(N-term)-mKate2. a, Super-resolution live two-colour time series of QL.p division in animals expressing the transgenic multicopy array *bcIs164*. Plasma membrane (myristoylated SFmTurquoise2ox) and chromatin (SFmTurquoise2ox::his-24) are shown in magenta, mitochondria (TOMM-20(N)-mKate2) in cyan. Images are maximum intensity projections of aligned z-stacks. In each image left= anterior side, right=posterior side, bottom= ventral side, top= dorsal side. **b**, Overview of QL.p cell and mitochondria 3D models in Imaris. **c**, Average TOMM-20(N)-mKate2 photobleaching from trends constructed on fluorescence values normalized to time 1 using unprocessed images. **d**, Mitochondrial quantities (post-cytokinesis) normalized to respective quantities at metaphase (*n* = 20, 24, 20). **c**, Average TOMM-20(N)-mKate2 photobleaching from trend constructed on fluorescence values normalized to time 1 (*n* = 12). P values are calculated using a Kruskal-Wallis non-parametric rank test with Benjamini, Krieger and Yekutieli correction (**d**). Normality was tested with the Shapiro-Wilk test. *: P-value ≤ 0.05; **: P-value ≤ 0.01; ***: P-value ≤ 0.001; ****: P-value ≤ 0.0001. Red bars in d = averages.

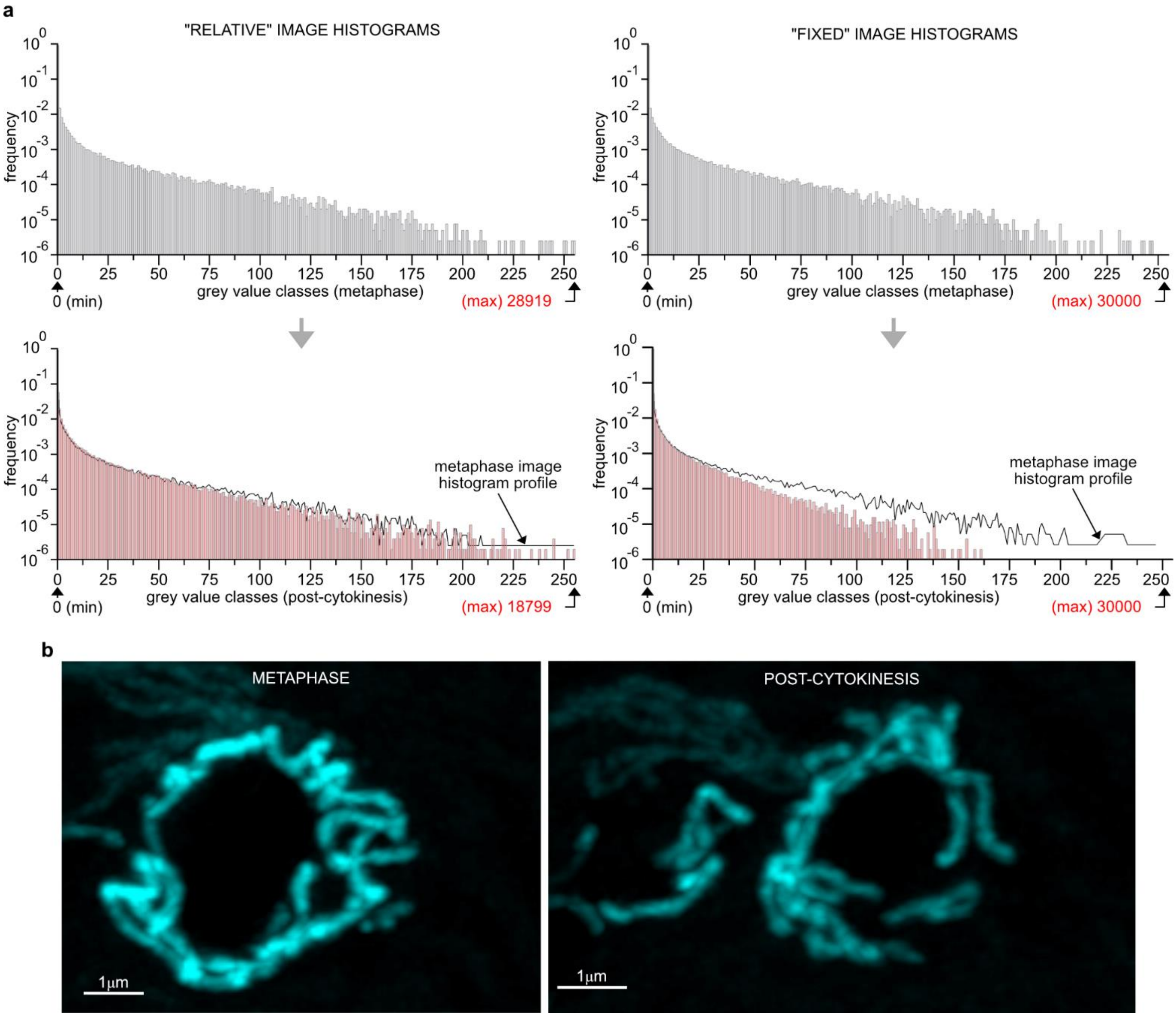

Fig.S9 | Image histograms: relative and fixed grey value classes. a, Image histograms, which represent the grey value frequency distributions across 256 classes spanning the min-max grey value range of z-stack mtGFP images, are generated from "fixed" or "relative" grey value ranges. The "relative" range (left) represents the interval, in which the max value is variable (image-specific) both between different time series and between metaphase (grey) and post-cytokinesis (red) images along the same time series. The "fixed" range (right) represents the 0-30000 grey value interval, which is invariant across all images. Histograms in this Figure were generated from fully processed images. **b**, *Maximum intensity* projection of aligned and image-subtracted metaphase and post-cytokinesis z-stacks (mtGFP) with brightness and contrast adjusted on the same grey value interval. The post-cytokinesis image (right) appears dimmer, due to fluorescence photobleaching. The two images refer to the histograms in Fig. S9a and they have been generated imaging QL.p cells in animals expressing *bcIs153*.

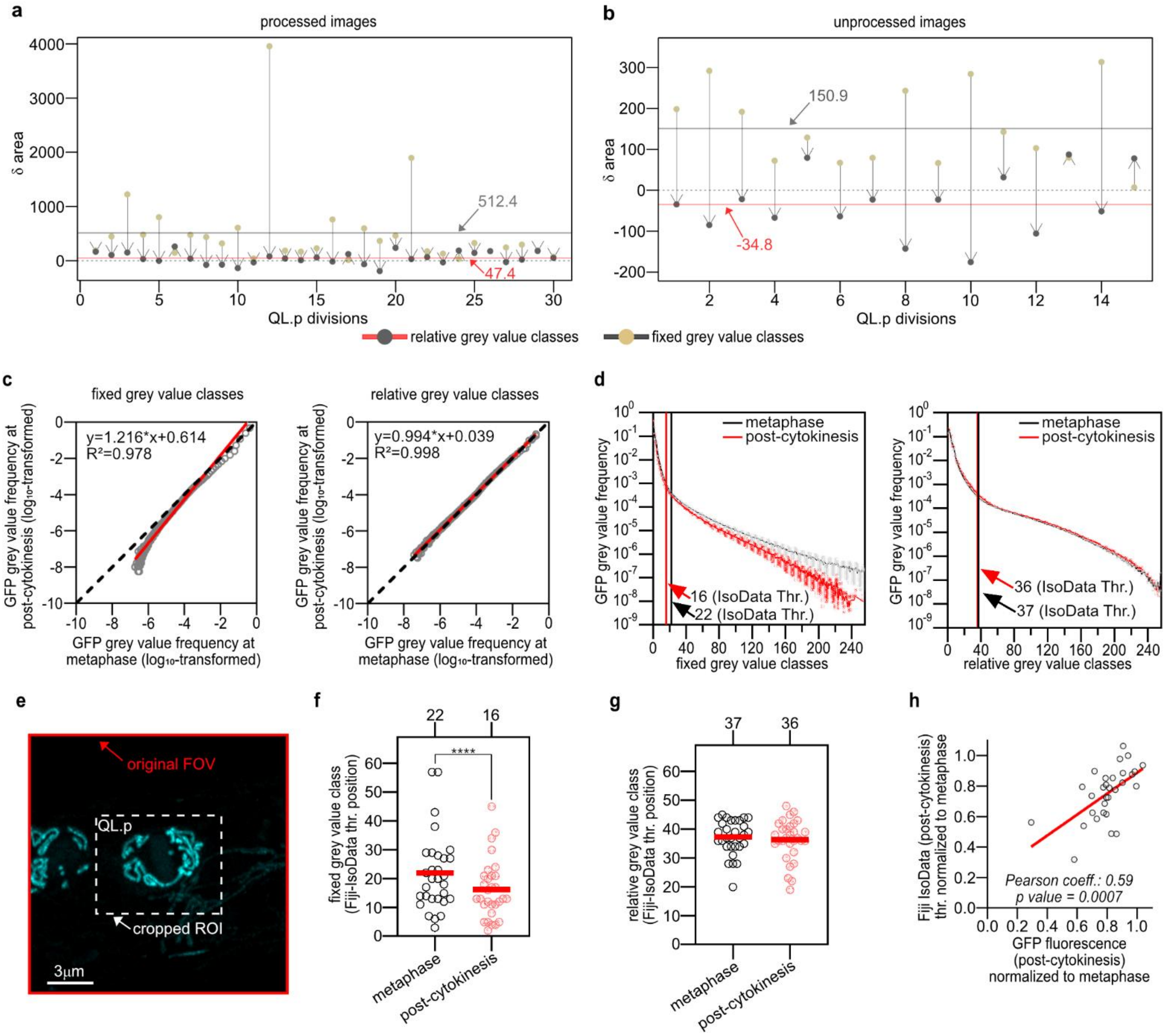

Fig.S10 | Photobleaching has minor effects on mtGFP grey value frequencies. a,b, Subtraction between metaphase and post-cytokinesis negative integrals (=δ area). Negative integrals were calculated from biexponential decay (processed images) and biexponential polynomial (unprocessed images) regressions of log transformed grey value frequencies measured on 256 fixed or relative grey value classes. Positive and negative y values refer to samples where metaphase regressions are above (i.e., right-shifted) and below (i.e., left-shifted) post-cytokinesis ones, respectively (see also supplementary information). Black arrows represent pairwise differences between relative and fixed values. Pairwise comparisons and one sample tests (**a**,**b**) were conducted between conditions and between each condition and the hypothetical 0 value, respectively (see main text). **c**, Linear regression of the relationship (see methods) between log-transformed average grey value frequencies measured on 256 fixed or relative grey value classes both at metaphase and post-cytokinesis from unprocessed images (see also Fig.S9 and Fig.S10e). **d**, Average mtGFP grey value frequency distributions generated on 256 fixed or relative grey value classes from unprocessed images (*n*=30) (see also Fig.S9) (error bars = SEM). **e**, Histograms from original mtGFP images (taken in *bcIs153*-expressing animals) were generated using original FOVs (red box) through whole z-stacks. **f**,**g**, Individual fixed or relative grey value classes to which Isodata (Fiji) threshold values belong, in unprocessed images (*n*=30). **h**, Correlation between normalized Isodata (Fiji) threshold values and normalized mtGFP fluorescence (photobleaching) at post-cytokinesis. P values are calculated using the Wilcoxon matched-pairs signed rank test (**f**,**g**), the One sample Wilcoxon test (**a**), or the One sampe t-test (**b**). Normality was tested with the Shapiro-Wilk test. P-value ≤ 0.05; **: P-value ≤ 0.01; ***: P-value ≤ 0.001; ****: P-value ≤ 0.0001 (**f**,**g**). Dotted lines represent the reference y=0 value (**a**,**b**). Red and black lines = mean (**a**,**b**). Red bars = mean (**f**,**g**).

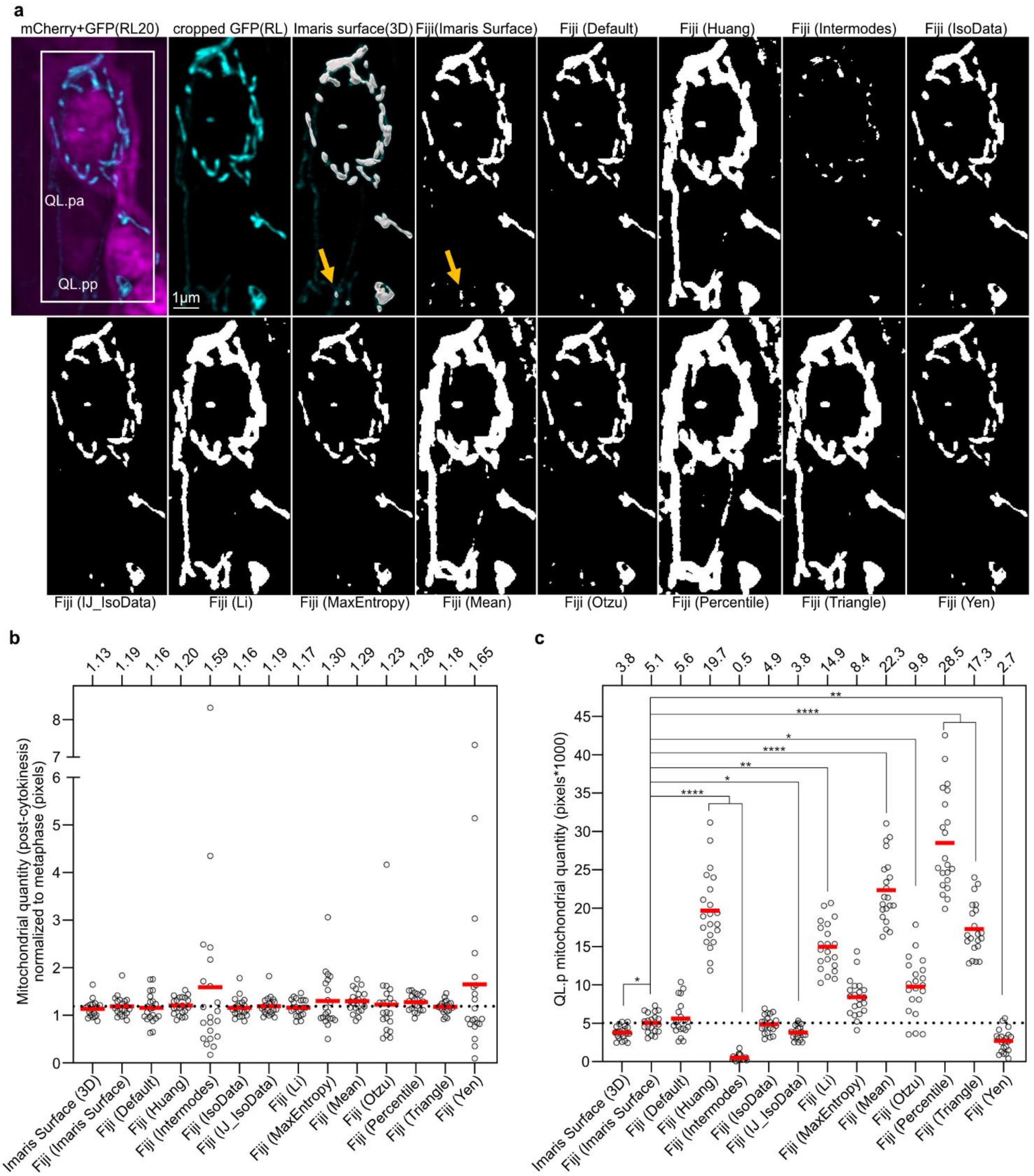

Fig.S11 | IsoData (Fiji) is the most comparable algorithm to Imaris Surface thresholding. a, Output binarization images from Fiji, generated using the same threshold values used for 3D rendering (*Fiji (Imaris Surface)*) and 12 Fiji global thresholds (images were generated imaging QL.p in animals expressing *bcIs153*). The "*Fiji (Imaris Surface)*" represents the control, to which all other Fiji thresholds are compared. The white box (top left image) and the "*cropped GFP (RL)*" represent the top view of the volume used to 3D render mitochondria in Imaris (see also Fig. 3c). The orange arrows indicate some mitochondria belonging to other cells that were included in Imaris 3D rendering and 2D binarizations and were not removed in this analysis. **b**, Mitochondrial quantities were normalized by calculating the post-cytokinesis-to-metaphase ratios (*n*=21). **c**, QL.p mitochondrial quantity is measured as the number of pixels composing the objects produced through image binarization (*n*=21). The voxel contents (*Imaris Surface (3D)*) match the same pixel contents, since each voxel in Imaris refer to the respective pixel of z-stack sections. **b**,**c**, the dotted lines represent the mean of *Fiji (Imaris Surface)* controls. P values are calculated using a non-parametric Friedman test with the Benjamini, Krieger and Yekutieli correction (**b**,**c**). Normality was tested with the Shapiro-Wilk test. *: P value ≤ 0.05; **: P value ≤ 0.01; ***: P value ≤ 0.001; ****: P value ≤ 0.0001. Red bars = mean.

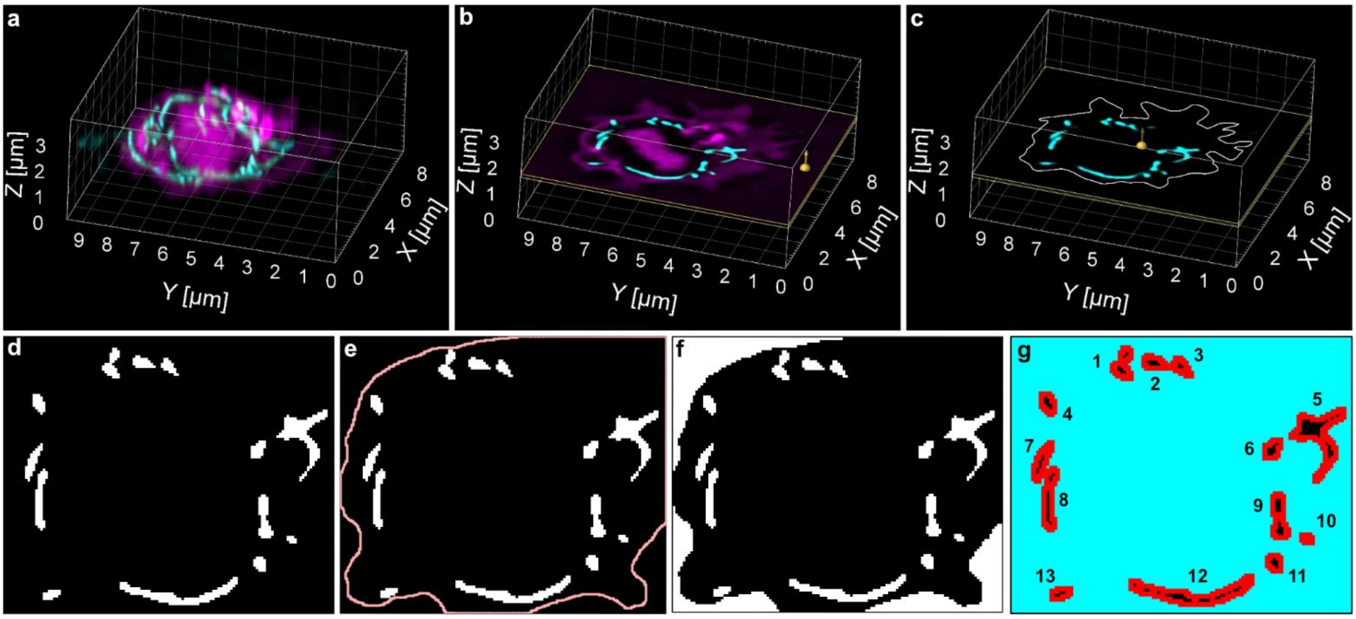

Fig.S12 | Workflow of a threshold-based segmentation (Fiji) comparable to Imaris Surface rendering. a, 3D view of processed z-stacks showing QL.p chromatin and plasma membrane (magenta) and mitochondria (cyan) at metaphase (in animals expressing *bcIs153*). **b**, Each plane sectioning QL.p (or QL.p daughters) across the z-stack is processed. **c**, Magenta and cyan images are split and only the cyan z-stack is used. **d**, Top view of the cyan z-stack (Fiji) representing the volume manually defined in Imaris to 3D render mitochondria. Automated global IsoData threshold was applied to the entire z-stack to generate a binary image highlighting 2D mitochondrial objects. **e**, ROIs matching the plasma membrane in Fiji (magenta image, same z-stack dimensions) were generated and applied on the binarized cyan z-stack. **f**, A macro running the clear function on inverse-made ROI selections deletes any object outside ROI selections in Fiji. **g**, New ROI lists are generated on the remaining 2D objects that belong to the cell of interest and can be measured individually in Fiji. **a**,**b**,**c** are illustrative representations in Imaris of the z-stack processed in Fiji.

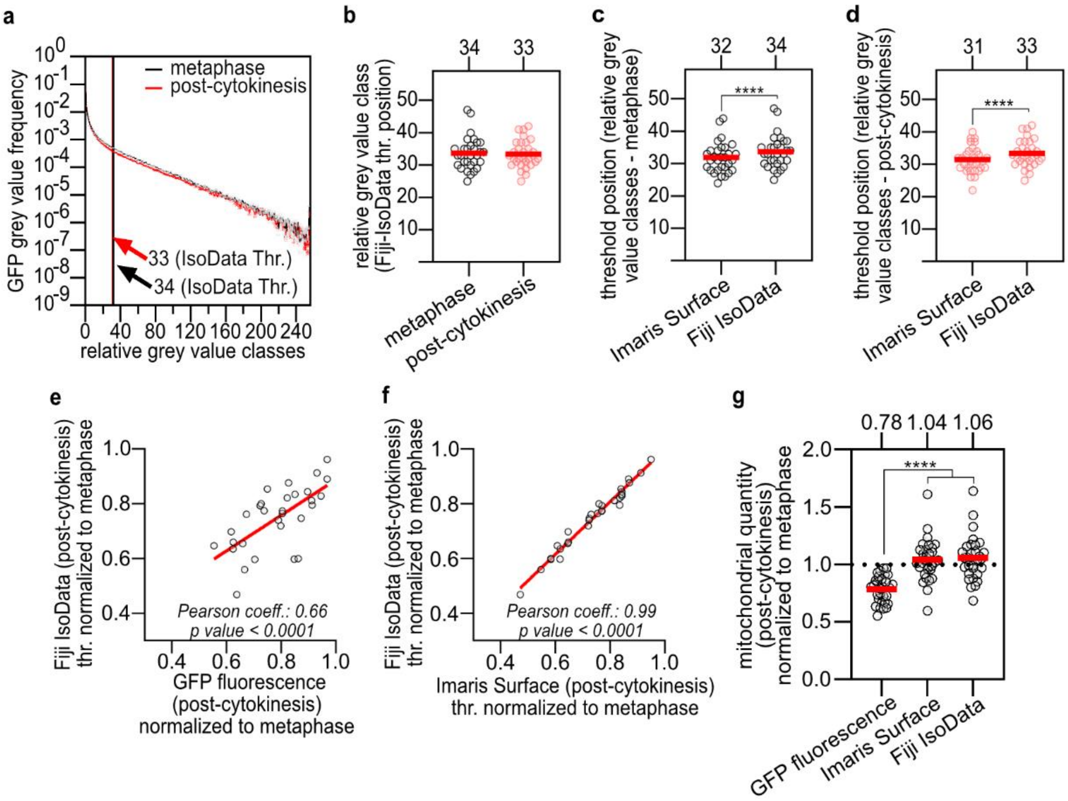

Fig.S13 | Photobleaching does not interfere with threshold-based segmentation of mitochondria in Fiji. a, Average mtGFP grey value frequency distributions generated on 256 relative grey value classes (*n*=30) (plot refers to Fig. 4g) (error bars = SEM). Mean grey value classes to which Fiji IsoData threshold values belong are showed. **b**, Individual relative grey value classes to which Fiji IsoData threshold values belong (*n*=30). **c**,**d**, Individual relative grey value classes to which Imaris Surface and Fiji IsoData threshold values belong, both a metaphase (left) and at post-cytokinesis (right) (*n*=30). **e**, Correlation between normalized Fiji IsoData threshold values and normalized mtGFP fluorescence (photobleaching) at post-cytokinesis. **f**, Correlation between normalized Imaris Surface threshold values and normalized Fiji IsoData threshold values. **g**, Mitochondrial quantities after division normalized to respective quantities at metaphase (*n*=30). All three datasets were generated on z-stacks representing the volumes manually defined to 3D render mitochondria in Imaris (see also Fig. 3c). *GFP fluorescence* and *Fiji IsoData* values were generated in Fiji, while *Imaris Surface* data were generated in Imaris. P values are calculated using a Wilcoxon matched-pairs signed rank test (**b**,**c**), a paired t test (**d**) or a Repeated Measures (RM) ANOVA with Geisser-Greenhouse correction and Benjamini, Krieger and Yekutieli correction (**g**). Normality was tested with the Shapiro-Wilk test. *: P value ≤ 0.05; **: P value ≤ 0.01; ***: P value ≤ 0.001; ****: P value ≤ 0.0001. Red bars = mean (**b**,**c**,**d** and **g**).

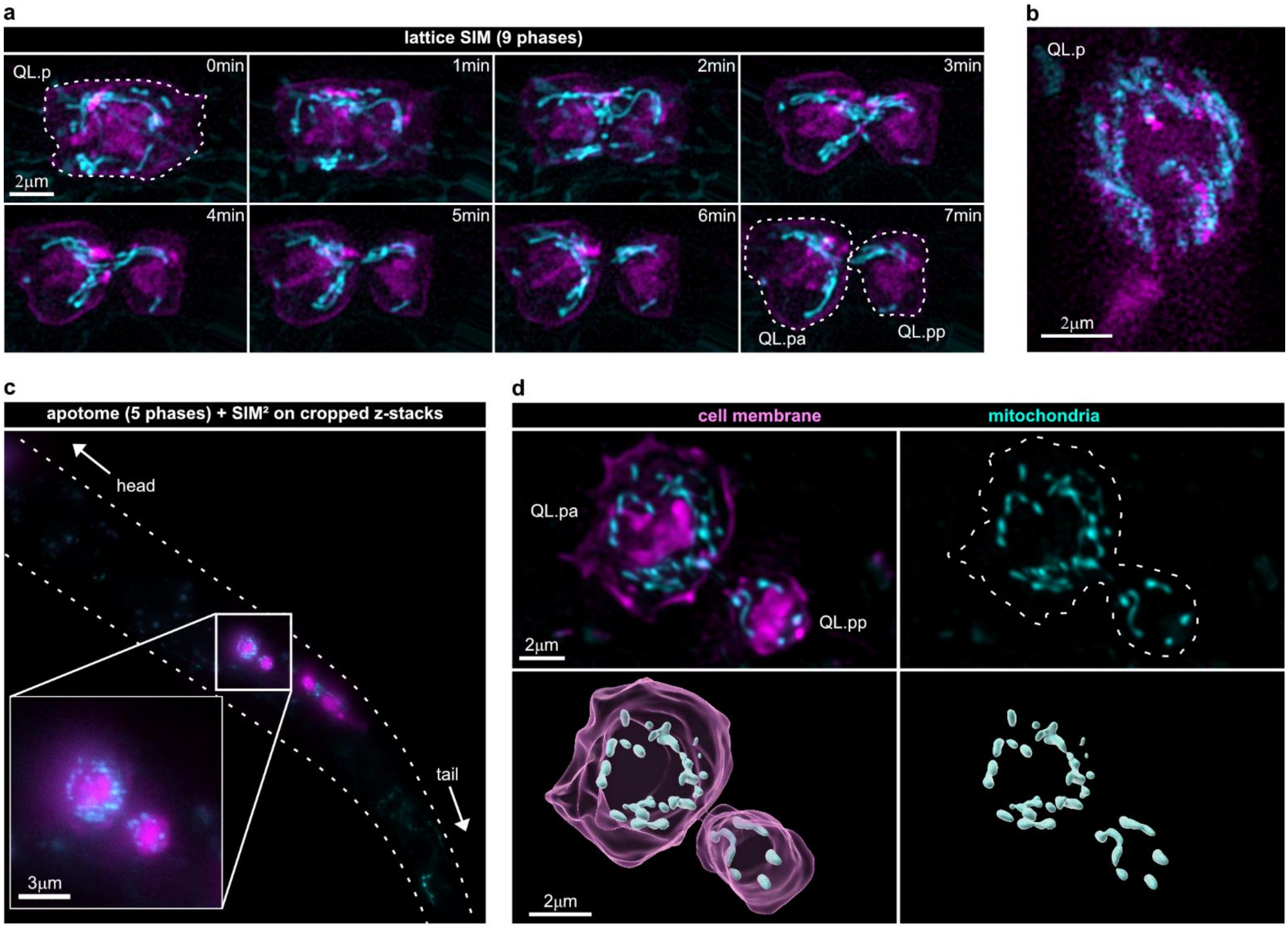

Fig.S14 | Mechanical immobilization of L1 larvae is compatible with Structured-Illumination Microscopy to achieve super resolution. a, Super-resolution live two-colour time series of QL.p division in animals expressing the transgenic multicopy array *bcls153* in lattice (9 phases) SIM mode. **b**, lattice SIM move is relatively slow and can cause motion blur in QL.p mitochondrial objects. **c**, fast acquisition of a large FOV (80µm*80µm*4.37µm) in apotome (5 phases) imaging mode. The white insert represents the selected 3D region that was processed thought the SIM^2 image reconstruction algorithm. **d**, super resolution output (the nominal image resolution in xy is 75nm) after SIM^2 image reconstruction of the 3D region highlighted by the insert in panel c. Top: fluorescence images; bottom: 3D rendered images. All images were generated using the Zeiss Elyra 7 SIM microscope upgraded with the SIM^2 image reconstruction algorithm.

Supplementary Movie 1 | XY change of QL.p position during z-stack acquisition. Single time point acquisition of QL.p during division in animals expressing the *bcIs153* transgene. Nanobeads-based mechanical immobilization effectively prevents animal movement during timeseries acquisitions, but occasional muscle contractions can still cause QL.p cell to move laterally (XY dimensions), relative to the optical axis. These movements do not normally cause obvious distortions of QL.p mitochondria.

Supplementary Movie 2 | XY change of QL.p position during timeseries acquisition. Timeseries acquisition of QL.p in animals expressing the *bcIs153* transgene. Nanobeads-based mechanical immobilization effectively prevents animal movement during timeseries acquisitions, but occasional muscle contractions can still cause QL.p cell to move. During this recording, the centre of the field of view was not changed. Acquisition settings are as described in methods, but acquiring images every 30 seconds.
