# Supplementary material for: Impact of photobleaching on quantitative, spatio-temporal, super-resolution imaging of mitochondria in live C. elegans larvae
Source: Npj Imaging. 2024 Nov 6;2:43. doi: 10.1038/s44303-024-00043-1 (PMC11541191; doi:10.1038/s44303-024-00043-1)
Supplement: Supplementary file 2 — Supplementary method [file 44303_2024_43_MOESM2_ESM.pdf]

Supplementary method

Image histogram data was acquired in dividing cells at metaphase and post-cytokinesis. Histogram data was used of both unprocessed and processed images (processing included z-stack alignment, image cropping, image subtraction, and image deconvolution). In order to analyse the data a constant equaling the smallest non-zero value was added to the frequency histogram data to deal with zeroes (a common statistical trick), after which it could be ln transformed (**Fig.S15**). This is because while zero can be approached, any logarithm of zero is undefined (depicted as `NaN` break in the y-axis at $y = 0$ in the left hand figure below). The zeroes become visible when the minimum value is added in the right hand figure below (blue ellipse). The ln transformation causes the data to be negative (notice y-axis) and allowed us to fit biexponential models to the data (e.g. dotted curves), with the grey area between providing a proxy of photobleaching that occurred.

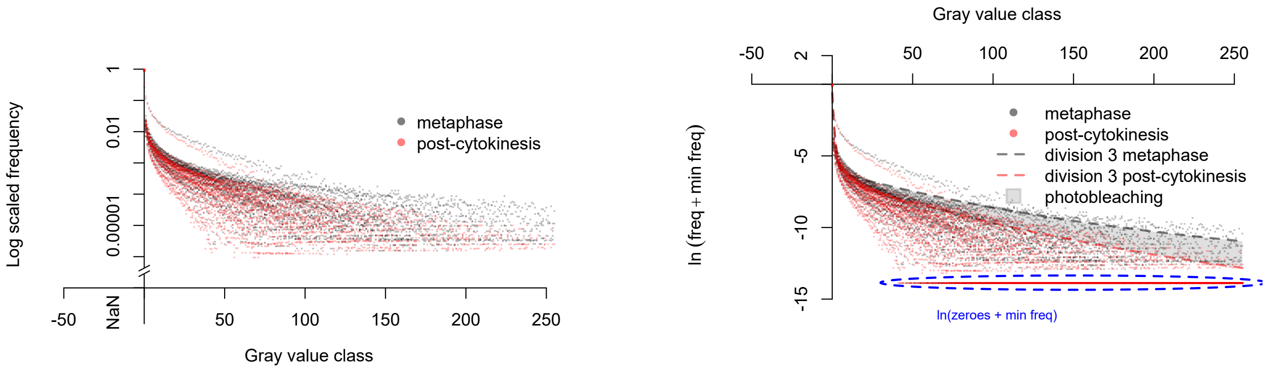

(a) Frequencies using a logarithmic scale. **(b)** Frequencies transformed using the natural logarithm.

Fig.S15 | Example histogram data and transformation.

Fitting these non-linear models to the data then allowed us to integrate underneath the curves for both stages of division (**Fig.S10 a,b**), with the area between the curves being a proxy of any photobleaching that may have occurred.

The robustness of relative thresholding to photobleaching can already be seen in figures (**Fig.S16**) below, where histogram data is condensed (less variance) under relative thresholding as opposed to fixed thresholding. The `LOESS` smoothed fits are also closer together for the relative thresholding datasets (right hand figures) compared to the fixed trhesholding datasets (left hand figures), for both the unprocessed and processed datasets. Also notice that images taken post-cytokinesis typically show more photobleaching (more red dots are “zeroes”). Relative thresholding then condenses metaphase and post-cytokinesis histogram data bringing the curves closer together, hence showing robustness to photobleaching

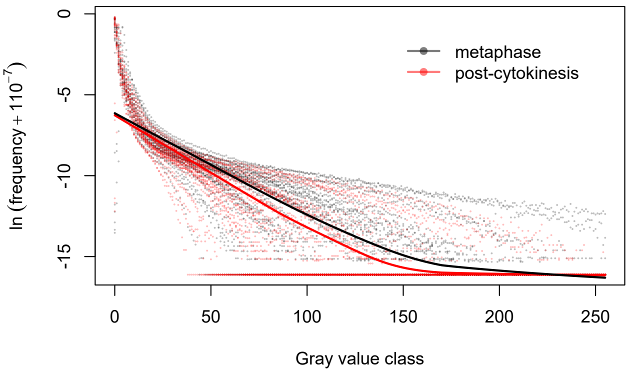

(a) Fixed thresholding in unprocessed images

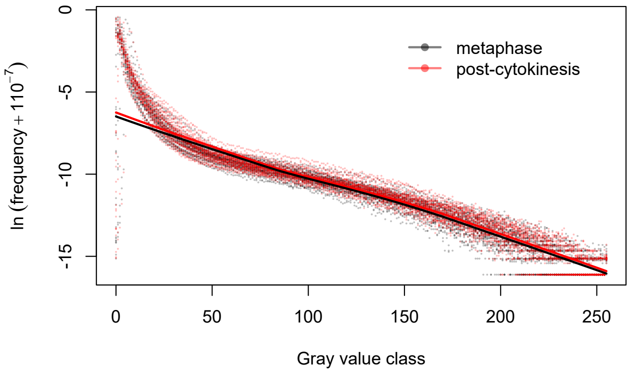

(b) Relative thresholding in unprocessed images

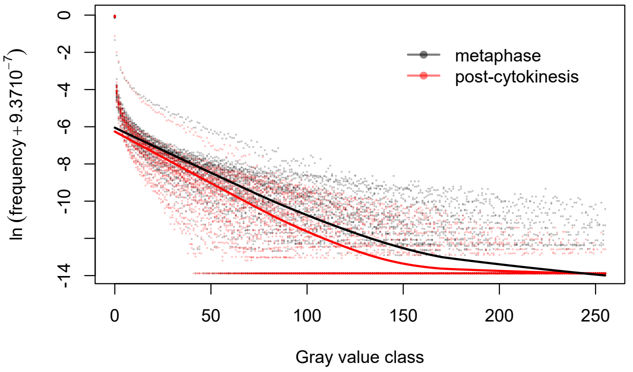

(c) Fixed thresholding in processed images

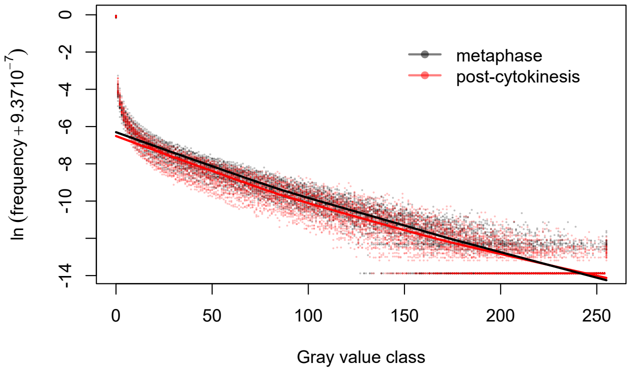

(d) Relative thresholding in processed images

Fig.S16 | In transformed histogram data with *fixed* and *relative* thresholding.

In order to estimate the robustness of relative thresholding to photobleaching, non-linear models were fitted using `nlme` (Pinheiro and Bates, 2000).

The data of the processed images were fitted to biexponential functions with an offset. The *fixed* image histogram data best fit:

$$ln(frequency + 9.37 * 10^{-7}) = \beta_1 \cdot e^{-\alpha_1 \cdot class} + \beta_2 \cdot e^{-\alpha_2 \cdot class} - \gamma$$

Whereas the histogram data with *relative* thresholding best fit with an intercept (γ) that was not allowed to vary:

$$ln(frequency + 9.37 * 10^{-7}) = \beta_1 \cdot e^{-\alpha_1 \cdot class} + \beta_2 \cdot e^{-\alpha_2 \cdot class} - 24.03$$

The definite integrals ($\int_0^{255}$) of the resulting curves, represent the area between the x-axis and the curve. By subtracting the areas above the curves before (metaphase) and after (post-cytokinesis) photobleaching, we acquired the area between the curves as a proxy for how close they are together. **Fig.S17-20** show the areas between the curves for both *fixed* (light grey) and *relative* (dark grey) thresholding for each division.

The δ area between the respective curves is shown in **Fig.S10 (a)**, with statistical tests given in the main text.

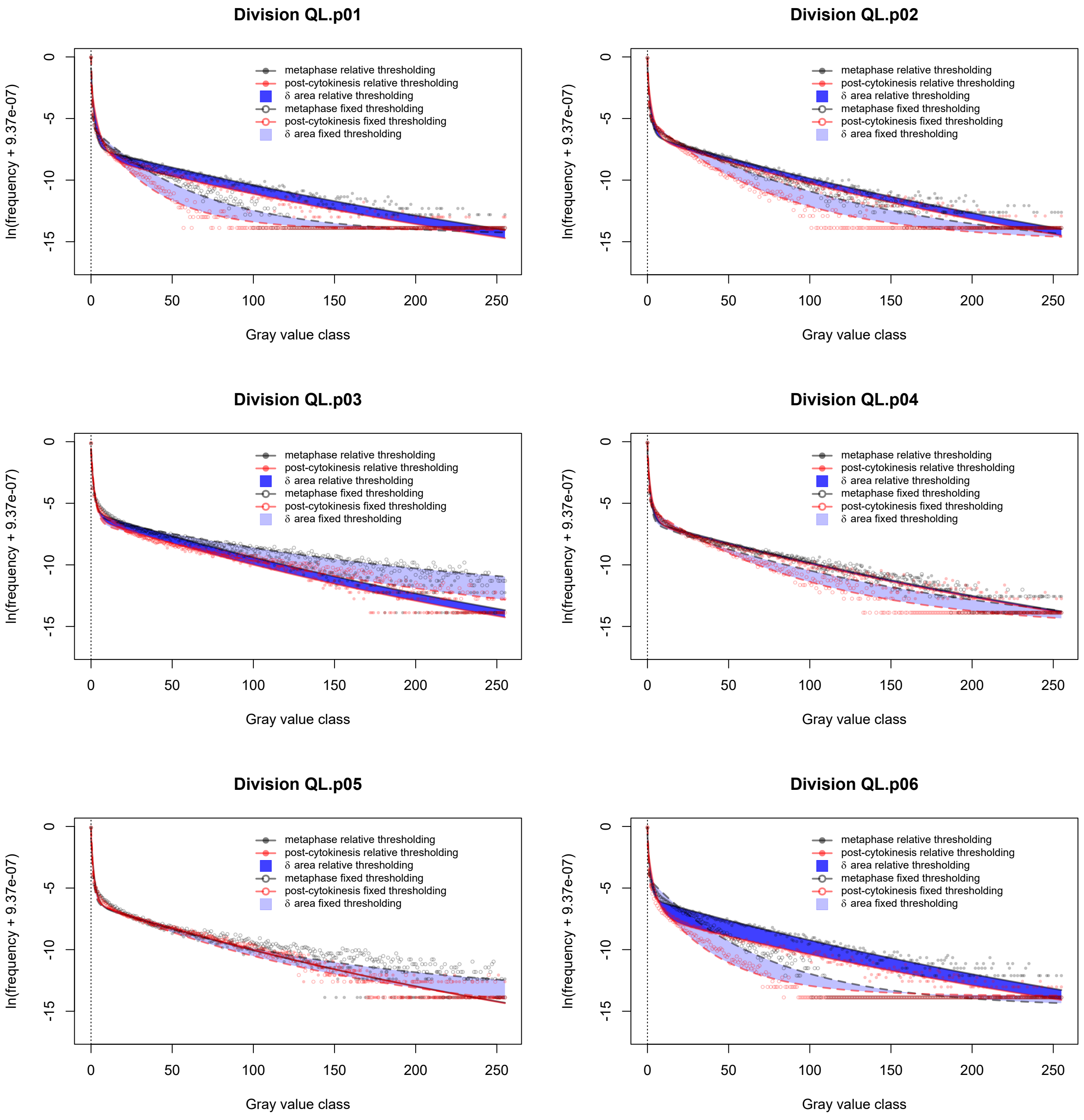

Fig.S17 | Fitted non-linear models for the processed image histogram data of divisions one through six.

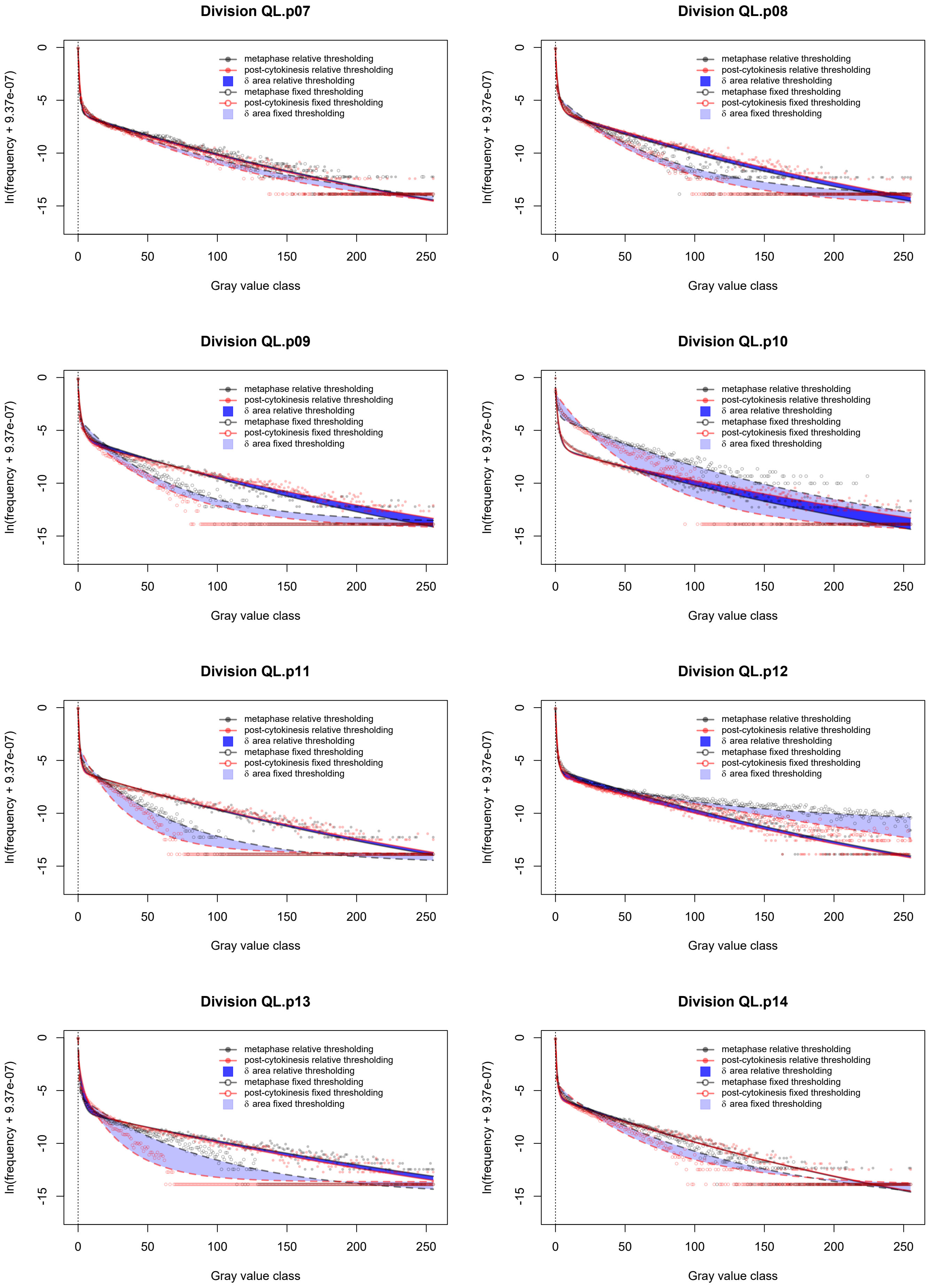

Fig.S18 | Fitted non-linear models for the processed image histogram data of divisions seven through fourteen.

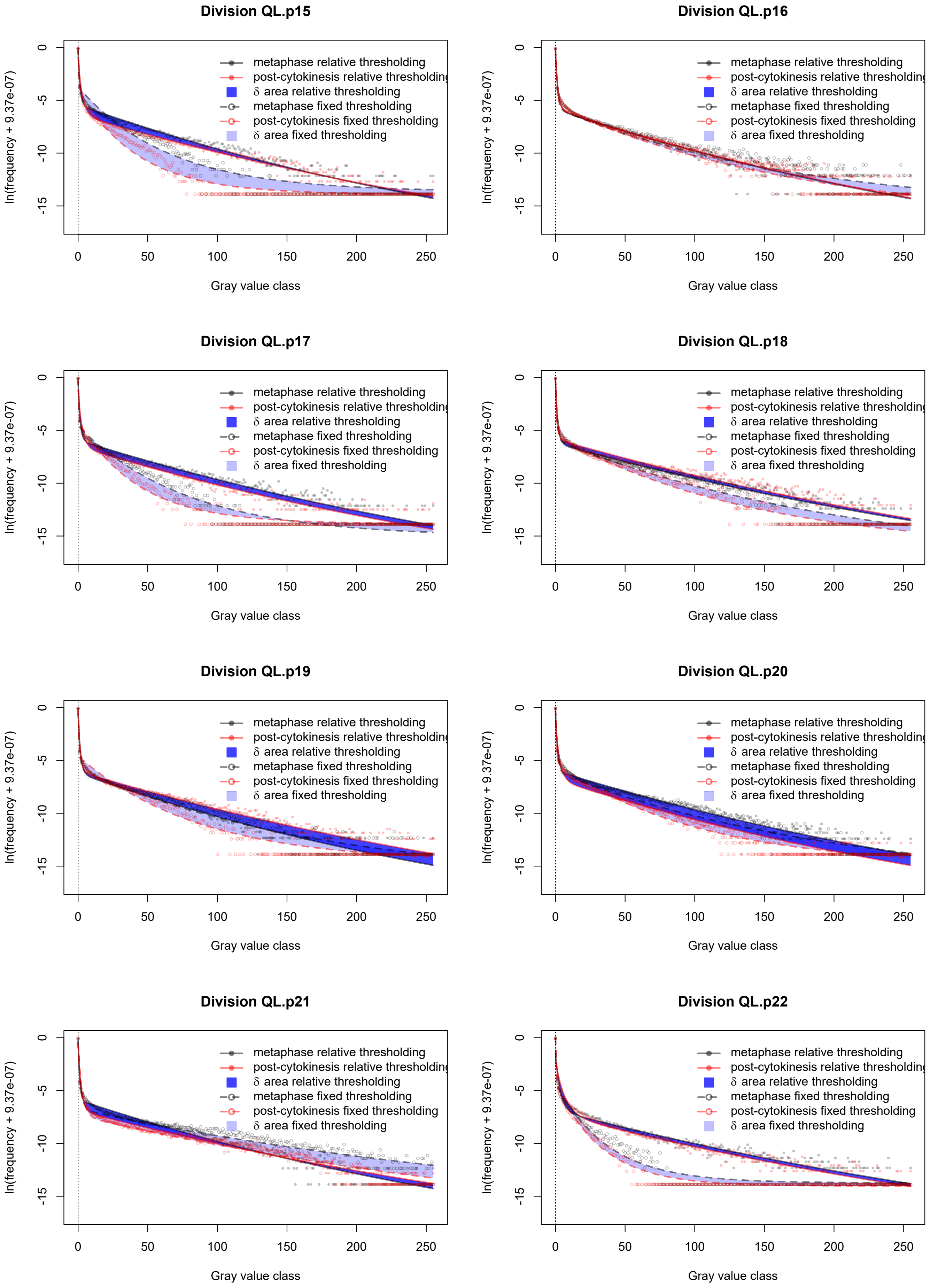

Fig.S19 | Fitted non-linear models for the processed image histogram data of divisions fifteen through twenty two.

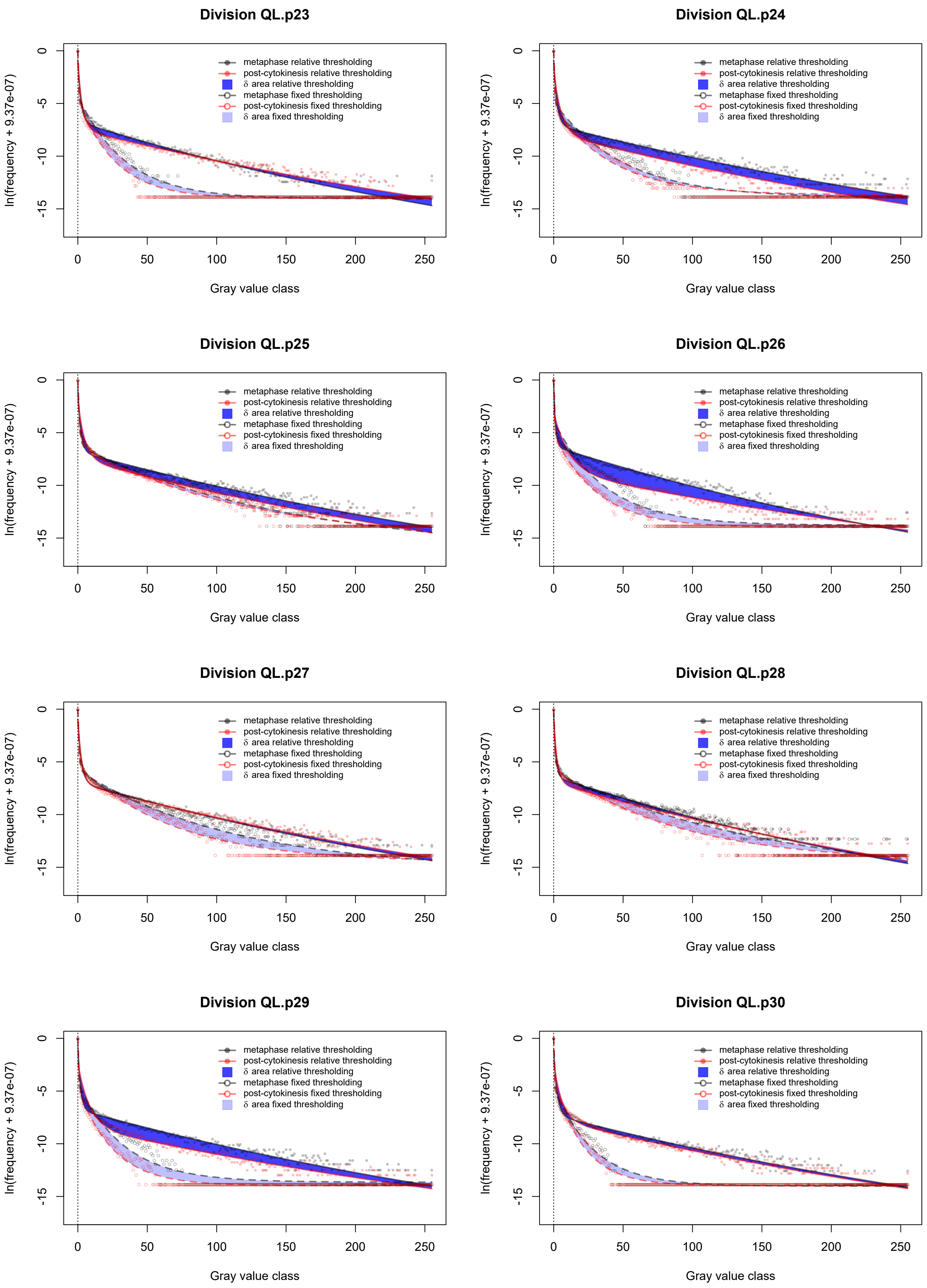

Fig.S20 | Fitted non-linear models for the processed image histogram data of divisions twenty three through thirty.

The preceding analysis was repeated for image histogram data of the unprocessed images. This data was best fit using biexponential polynomials. The fixed thresholding histogram data best fit:

$$ln(frequency) = \beta_1 \cdot e^{-\alpha_1 \cdot class} + 22.54 \cdot e^{-\alpha_2 \cdot class} - \beta_2 \cdot class - \gamma$$

The relative thresholding histogram data best fit:

$$ln(frequency) = \beta_1 \cdot e^{-\alpha_1 \cdot class} + 7.7 \cdot e^{-\alpha_2 \cdot class} - \beta_2 \cdot class - \gamma$$

Fits were much harder to obtain for the unprocessed images, and parameter estimates for all curves could only be obtained for fifteen divisions in total (**Fig.S21-23**). Likewise δ area between the respective curves is shown in **Fig.S10 (b)**, with statistical tests given in the main text.

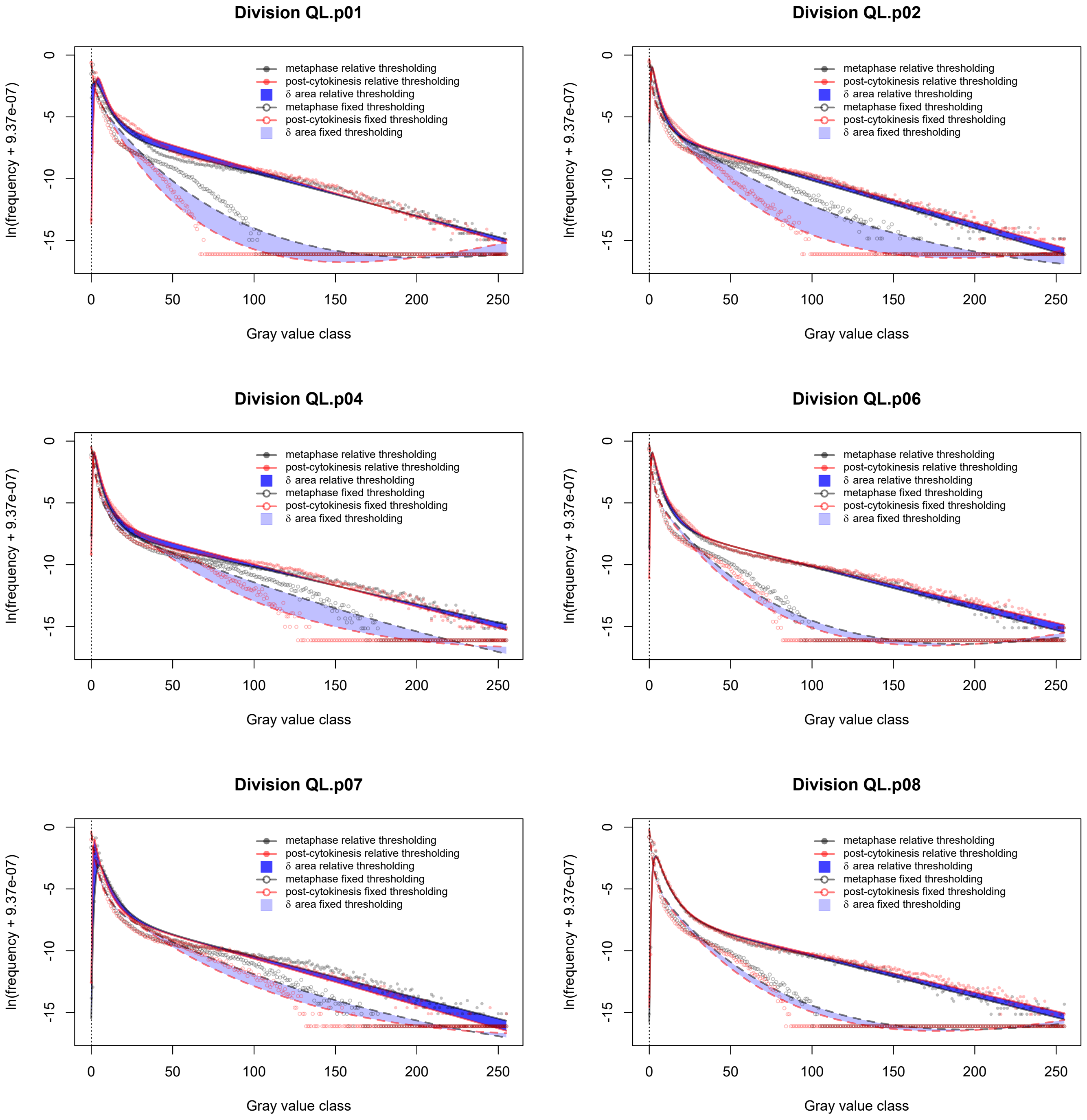

Fig.S21 | Fitted non-linear models for the unprocessed image histogram data of divisions one, two, four, six, seven and eight

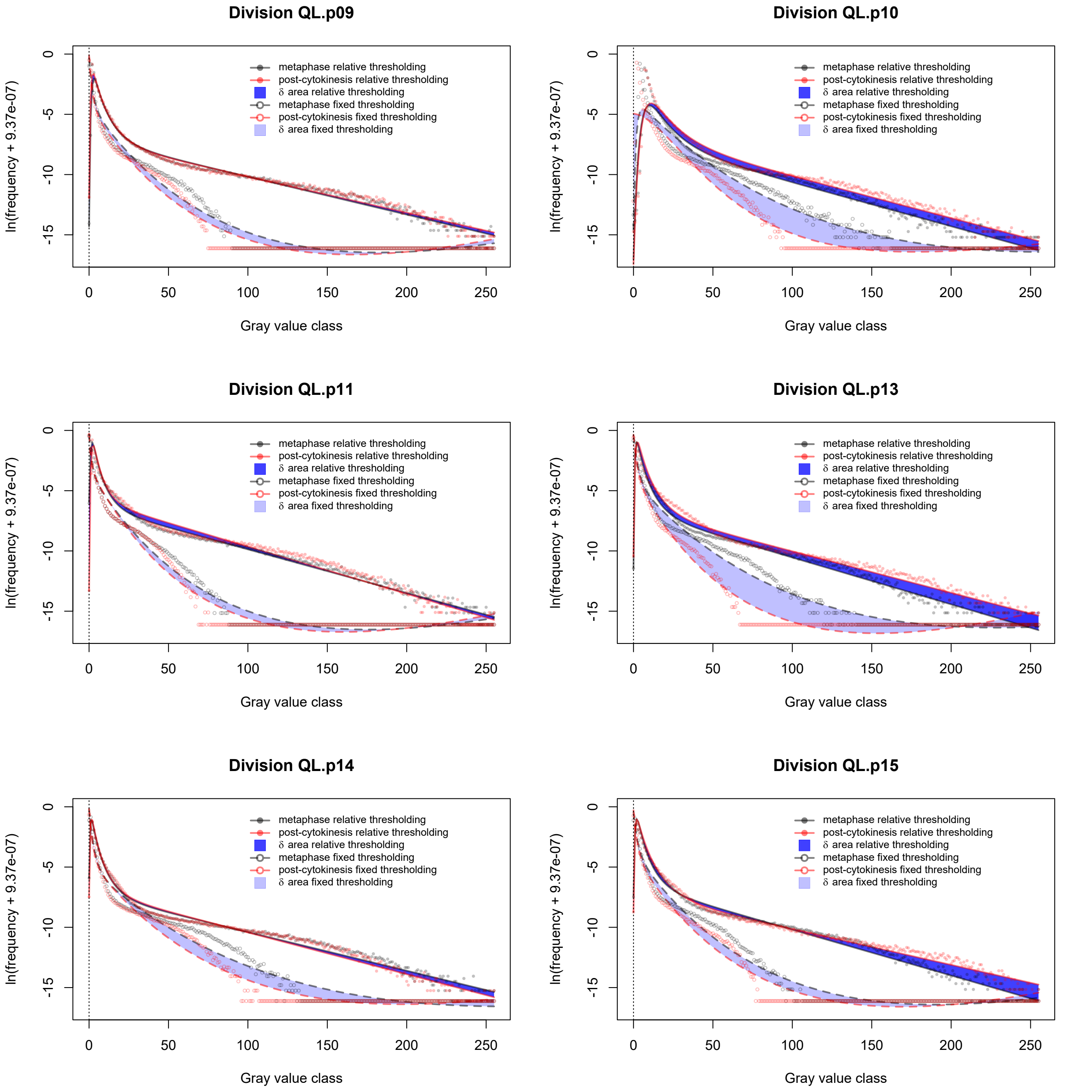

Fig.S22 | Fitted non-linear models for the unprocessed image histogram data of divisions nine through eleven, and thirteen through fifteen.

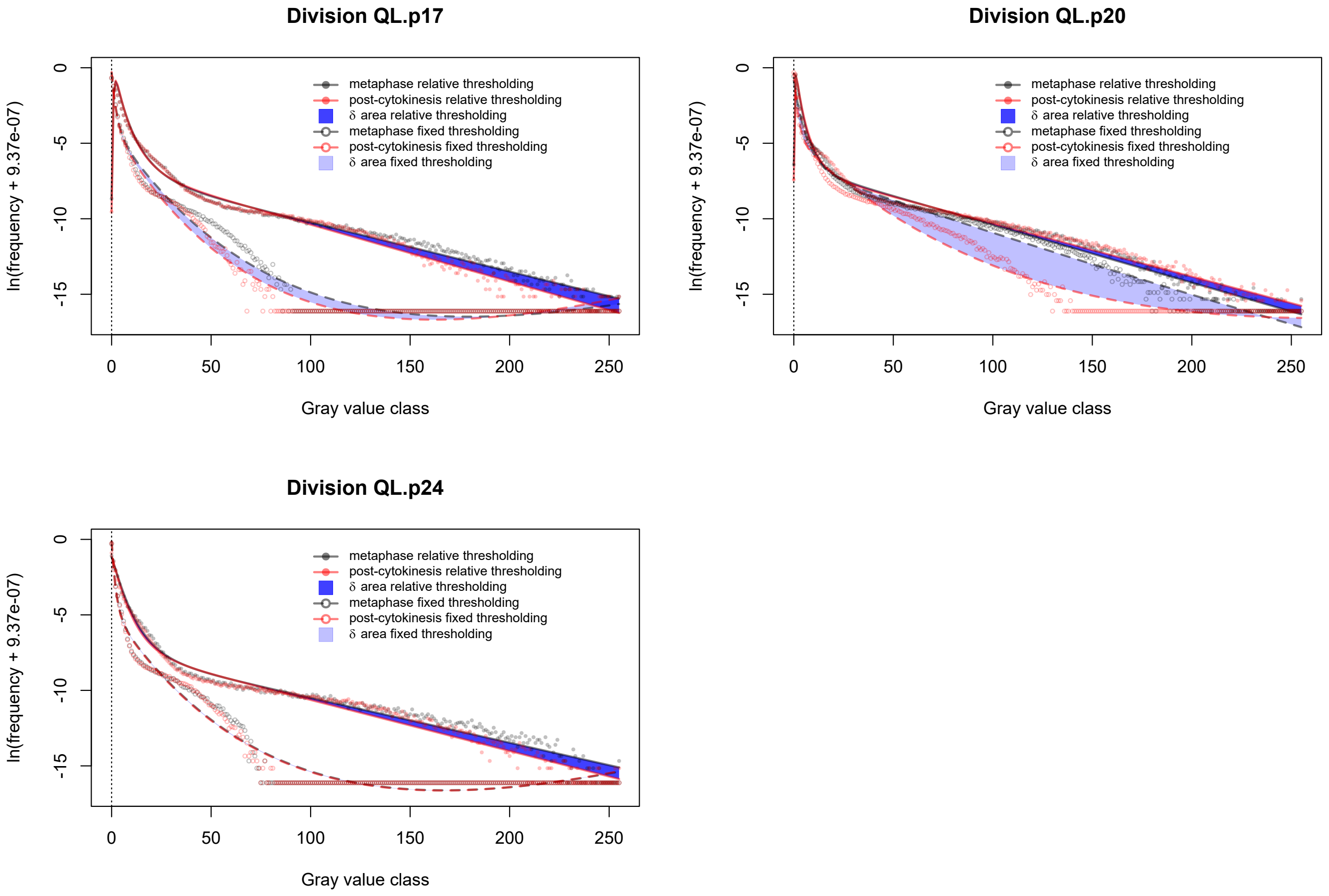

Fig.S23 | Fitted non-linear models for the unprocessed image histogram data of divisions seventeen, twenty and twenty four.

References

Pinheiro, J.C., and Bates, D.M. (2000) “Mixed-Effects Models in S and S-PLUS”, Springer.
